# Neutrophil CEACAM1 determines susceptibility to NETosis by regulating the S1PR2/S1PR3 axis in liver transplantation

**DOI:** 10.1172/JCI162940

**Published:** 2023-02-01

**Authors:** Hirofumi Hirao, Hidenobu Kojima, Kenneth J. Dery, Kojiro Nakamura, Kentaro Kadono, Yuan Zhai, Douglas G. Farmer, Fady M. Kaldas, Jerzy W. Kupiec-Weglinski

**Affiliations:** 1 Dumont-UCLA Transplantation Center, Department of Surgery, Division of Liver and Pancreas Transplantation, David Geffen School of Medicine at UCLA, Los Angeles, California, USA.; 2 Department of Surgery, Division of Hepato-Biliary-Pancreatic Surgery and Transplantation, Department of Surgery, Graduate School of Medicine, Kyoto University, Kyoto, Japan.; 3 Department of Pathology and Laboratory Medicine, David Geffen School of Medicine at UCLA, Los Angeles, California, USA.

**Keywords:** Transplantation, Hypoxia, Neutrophils, Organ transplantation

## Abstract

Neutrophils, the largest innate immune cell population in humans, are the primary proinflammatory sentinel in the ischemia-reperfusion injury (IRI) mechanism in orthotopic liver transplantation (OLT). Carcinoembryonic antigen–related cell adhesion molecule 1 (CEACAM1, *CC1*, or *CD66a*) is essential in neutrophil activation and serves as a checkpoint regulator of innate immune-driven IRI cascade in OLT. Although *CC1* alternative splicing generates two functionally distinct short and long cytoplasmic isoforms, their role in neutrophil activation remains unknown. Here, we undertook molecular and functional studies to interrogate the significance of neutrophil CC1 signaling in mouse and human OLT recipients. In the experimental arm, we employed a mouse OLT model to document that ablation of recipient-derived neutrophil CC1-long (CC1-L) isotype aggravated hepatic IRI by promoting neutrophil extracellular traps (NETs). Notably, by regulating the S1P–S1PR2/S1PR3 axis, neutrophil CC1-L determined susceptibility to NET formation via autophagy signaling. In the clinical arm, liver grafts from 55 transplant patients selectively enriched for neutrophil CC1-L showed relative resistance to ischemia-reperfusion (IR) stress/tissue damage, improved hepatocellular function, and clinical outcomes. In conclusion, despite neutrophils being considered a principal villain in peritransplant tissue injury, their CC1-L isoform may serve as a regulator of IR stress resistance/NETosis in human and mouse OLT recipients.

## Graphical abstract

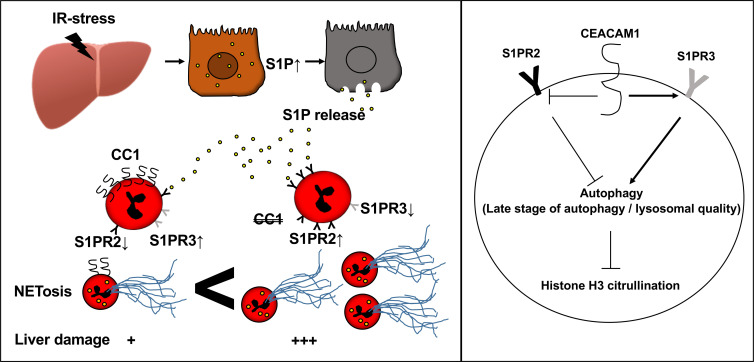

## Introduction

Orthotopic liver transplantation (OLT) is the mainstay of treatment for patients with end-stage liver disease and certain hepatic malignancies. However, ischemia-reperfusion injury (IRI), a consequence of donor liver procurement and transplantation, results in an innate immune-driven sterile inflammation that contributes to OLT dysfunction, a higher incidence of rejection episodes, and a shortage of life-saving donor organs (1). Even though preventing liver IRI is essential for improved clinical OLT outcomes, its underpinning mechanisms remain to be determined.

Carcinoembryonic antigen–related cell adhesion molecule 1 (CEACAM1, *CC1*, or *CD66a*), a transmembrane biliary glycoprotein, is expressed on epithelial, endothelial, and immune cells. The *CC1* gene undergoes alternative splicing to generate functionally distinct short and long isoforms (2). The CC1-short (CC1-S) variant (for its *short* 12–amino acid tail) associates with epithelial cells and regulates mucosal immunity. In contrast, the CC1-long (CC1-L) (for its *long* >70+ amino acid tail) isoform produces inhibitory signaling in myeloid and lymphocytic cells (3). We have identified CC1 as a checkpoint regulator of sterile IRI in mouse and human OLT by showing its expression dictated donor liver quality and prevented IRI by suppressing the ASK1/phosphorylated p38 (p-p38) cell death pathway (4). While hepatocyte CC1-S isoform may regulate hepatoprotection (4), the role of CC1-L signaling in OLT remains unknown.

CC1 is highly expressed on the surface of neutrophils, the largest innate immune cell population in humans, serving as the first line of defense against exogenous pathogens (1). Activated neutrophils, the main amplifiers of the hepatic IRI immune cascade, can release neutrophil extracellular traps (NETs), a newly identified lytic cell death pathway, to trap and kill extracellular microbes via a cellular process called NETosis. Much as NET formation is critical for host defense against pathogens, NETosis can cause tissue damage (5) by releasing damage-associated molecular patterns (DAMPs), such as histones and high mobility group box 1 (HMGB1), which stimulate macrophage activation to elaborate cytokines and facilitate sterile inflammation response (6, 7). In addition, NETs have been implicated in the pathogenesis of autoimmune diseases, metabolic disorders, and cancer (8–10).

While crosstalk between NETs and CC1 has been investigated in cancer research, there is limited knowledge of the relationship between neutrophil CC1 and NET generation. Rayes et al. demonstrated that, despite CC1 on NETs associated with tumor progression, CC1 itself was dispensable for NET formation in vitro (11). In contrast, nonalcoholic steatohepatitis (NASH) studies have revealed that neutrophils undergo NETosis through the interaction of sphingosine-1-phosphate (S1P), a bioactive signaling lipid, with its extracellular cognitive G protein–coupled receptor ligands (S1PR1–5), four of which (S1PR1–4) associate with neutrophil activation (12, 13). Despite the essential function of S1P and S1PR1–5 in neutrophil development, whether and how CC1 coordinates this activity remain to be elucidated. Given CC1 has been closely linked with the development of NASH (14), we reasoned neutrophil CC1 might be involved in the S1P/S1PRs signaling pathway.

This translational study documents the benefits of neutrophil CC1-L to the susceptibility of donor livers to peritransplant stress in mouse and human OLT recipients. First, we employed a murine OLT model to identify recipient-derived neutrophil CC1-L signaling–attenuated hepatic IRI. Notably, by regulating the S1PR2/S1PR3 axis, neutrophil CC1-L suppressed NET formation via the autophagy pathway. In the clinical arm, 55 patients with liver transplants enriched in neutrophil CC1-L showed ischemia-reperfusion (IR) stress resistance, leading to improved hepatocellular function and clinical outcomes. Thus, despite being recognized as a principal villain in tissue injury, neutrophil CC1-L isoform may serve as a regulator of IR stress and NETosis in OLT recipients.

## Results

### Recipient-derived neutrophils are the primary source of CC1-L isoforms in mouse OLT.

**Figure 1:**
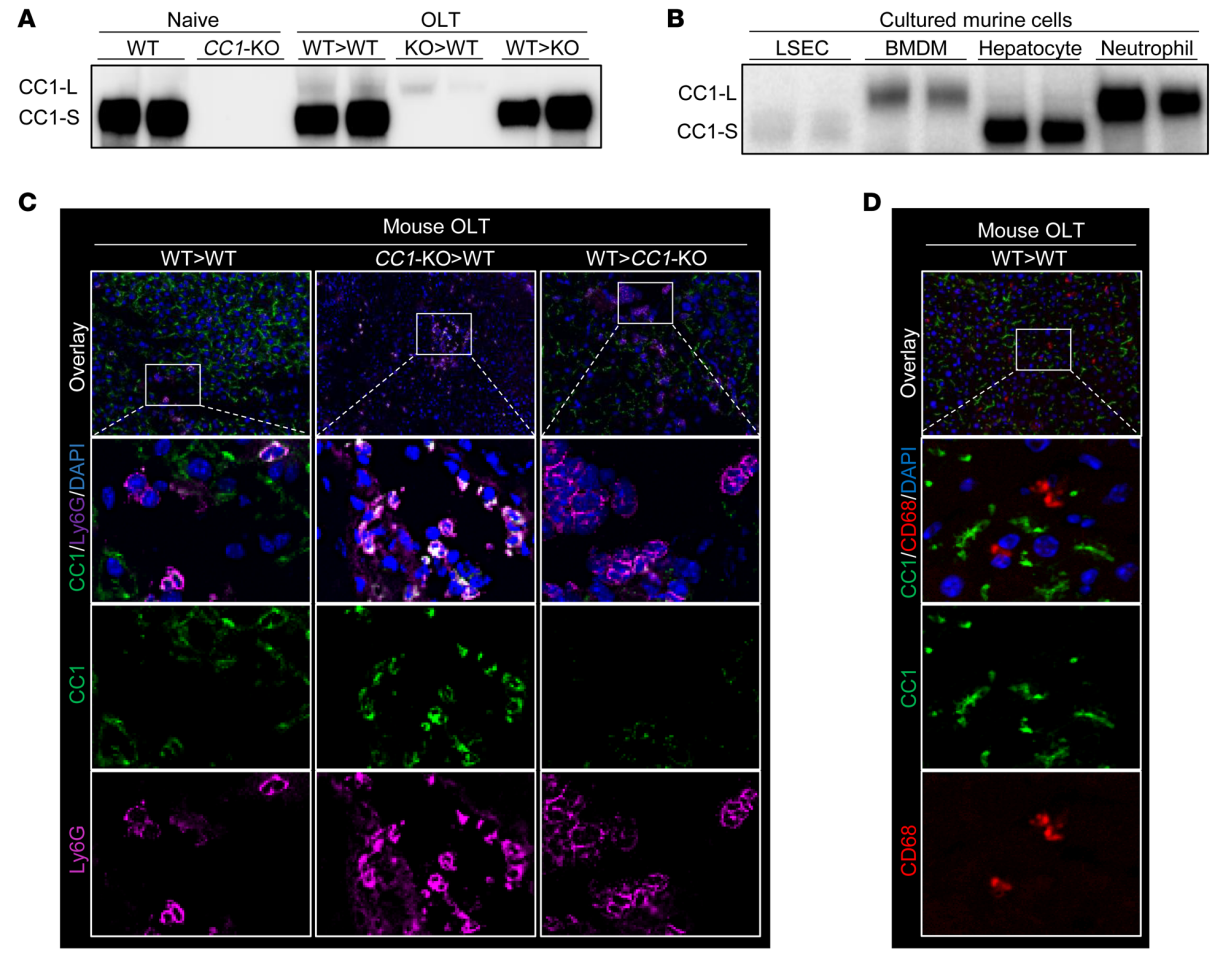
Recipient-derived CC1-L–expressing neutrophils infiltrate mouse OLT. (**A**) Mouse donor livers, stored in UW solution (4°C/18 hours), were transplanted into groups of WT and *CC1*-KO recipients, and OLT samples were collected at 6 hours after reperfusion. WB of CC1-S and CC1-L in naive and posttransplant livers (WT→WT, *CC1*-KO→WT, WT→*CC1*-KO). (**B**) WB of CC1-S and CC1-L in cultured murine cells (LSECs, BMDMs, hepatocytes, and neutrophils). (**C**) Representative (*n* = 3) IF images of CC1 (green), Ly6G (purple), and DAPI (blue) in OLT (WT→WT, *CC1*-KO→WT, WT→*CC1*-KO). Original magnification, ×200. (**D**) Representative (*n* = 3) IF images of *CC1* (green), CD68 (red), and DAPI (blue) in OLT (WT→WT). Original magnification, ×200 (top rows); ×1200 (bottom 3 rows).

We used our established mouse OLT model, in which donor livers, stored in a cold UW solution (4°C/18 hours), were transplanted into syngeneic recipients (WT→WT, *CC1*-KO→WT, WT→*CC1*-KO). Liver grafts were collected at 6 hours after reperfusion, the peak of the hepatocellular damage in this model (4), and screened for CC1 expression by Western blot (WB) (Figure 1A). In contrast with naive (WT) livers, which exclusively expressed the CC1-S isoform, in livers transplanted into WT mouse recipients (WT→WT and *CC1*-KO→WT), the CC1-L isoform was selectively detected. Indeed, livers implanted into *CC1*-deficient hosts (WT→*CC1*-KO) failed to express CC1-L despite proficient CC1-S levels, indicating that the CC1-L isoform in IR-stressed OLT derived from recipient-originating circulating cells.

We then analyzed the expression of CC1 isoforms in murine cell cultures (Figure 1B). Liver sinusoidal endothelial cells (LSECs) and hepatocytes expressed predominantly CC1-S, while bone marrow–derived macrophages (BMDMs) and neutrophils (Ly6G^+^) expressed abundant CC1-L isoforms. Immunoblots of CC1 isoforms/loading controls in cultured cells are shown (Supplemental Figure 1; supplemental material available online with this article; https://doi.org/10.1172/JCI162940DS1).

To identify the cellular origin of CC1-L in OLT, we employed immunofluorescence (IF) staining (Figure 1C). Unlike OLT-infiltrating Ly6G^+^ neutrophils exhibiting robust cytoplasmic CC1 in WT hosts (WT→WT and *CC1*-KO→WT), there was no Ly6G^+^*CC1* expression in livers transplanted into *CC1*-null mice (WT→*CC1*-KO). As intragraft CD68^+^ cells expressed relatively low CC1 levels (Figure 1D), we concluded that CC1-L isoform is confined primarily to recipient-derived OLT-infiltrating neutrophils.

### Recipient CC1 ablation exacerbates hepatic IRI in mouse OLT.

**Figure 2:**
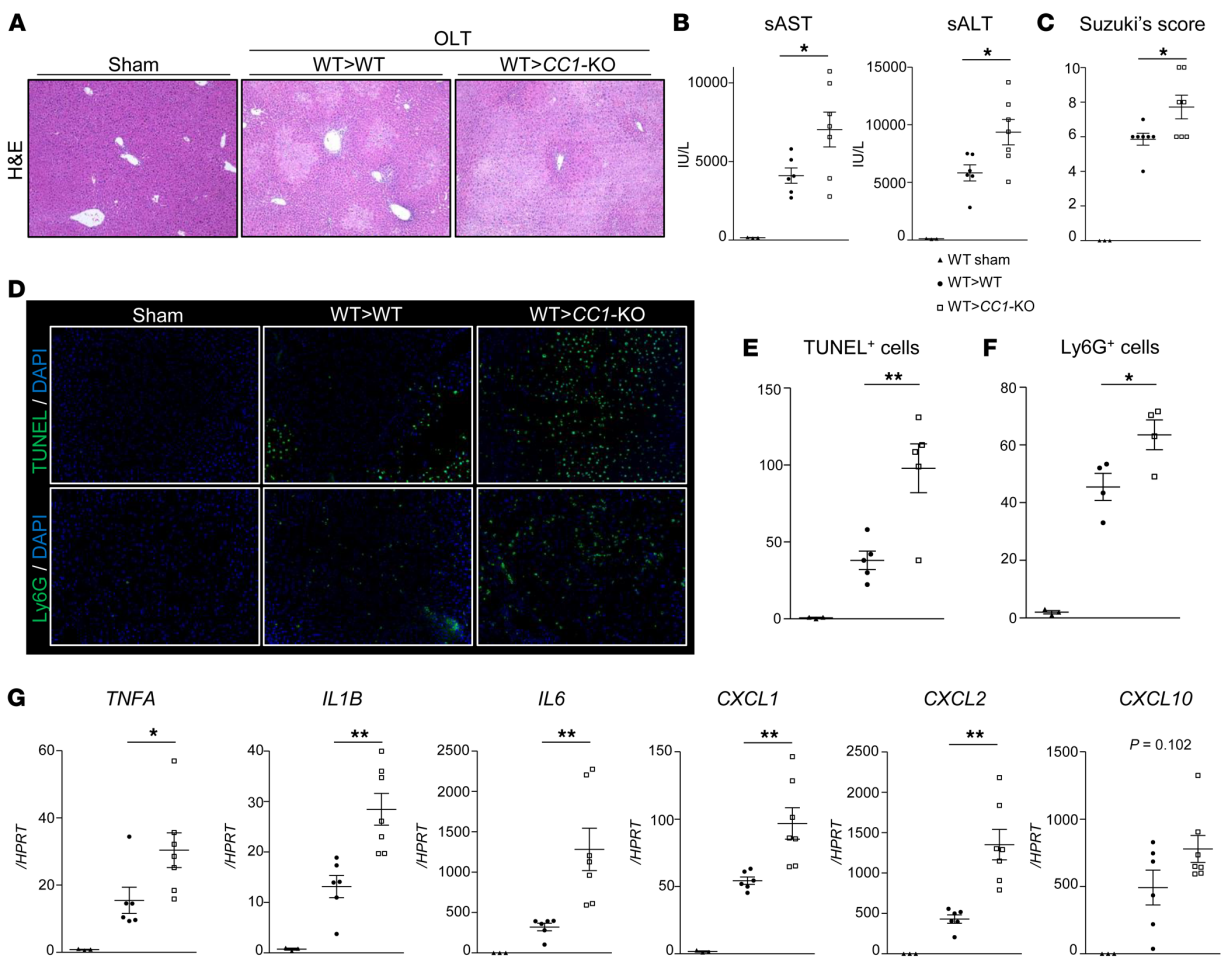
Recipient *CC1*-null mutation exacerbates hepatocellular damage and inflammatory response in IR-stressed mouse OLT. WT donor livers, stored in UW solution (4°C/18 hours), were transplanted into WT or *CC1*-KO recipients, and OLT samples were collected at 6 hours after reperfusion. (**A**) Representative H&E staining of sham-treated livers and OLT (WT→WT, WT→*CC1*-KO). Original magnification, ×100. (**B**) sAST and sALT levels (IU/L). (**C**) Suzuki’s histological grading of liver IRI. (**D**) Representative TUNEL and IF images of OLT-infiltrating Ly6G^+^ cells/field. Original magnification, ×200. (**E** and **F**) Quantification of TUNEL^+^ cells and Ly6G^+^ cells. (**G**) qRT-PCR–assisted detection of mRNA coding for TNFA, IL1B, IL6, CXCL1, CXCL2, and CXCL10. Data were normalized to HPRT gene expression. *n* = 6–7/group (**A**–**C** and **G**); *n* = 4–5/group (**D**–**F**). Data are represented as mean ± SEM. *P < 0.05; **P < 0.01, 1-way ANOVA followed by Tukey’s HSD test.

We next aimed to determine the impact of CC1 signaling on the hepatocellular damage in cold-stored (4°C/18 hours) livers transplanted into WT and *CC1*-KO recipients. At 6 hours after OLT, WT livers implanted into *CC1*-KO mice showed increased sinusoidal congestion, edema vacuolization, and hepatocellular necrosis (Figure 2A), enhanced serum aminotransferase (sAST) and alanine aminotransferase (sALT) release (Figure 2B), higher Suzuki’s histological score of IR damage (Figure 2C), increased frequency of TUNEL^+^ and Ly6G^+^ cells (Figure 2, D and E), and enhanced hepatic mRNA levels coding for TNFA, IL1B, IL6, CXCL1, CXCL2, and CXCL10 (Figure 2G) compared with those in *CC1*-proficient counterparts (WT→WT). These results suggest that recipient-derived CC1 may be beneficial in mitigating innate immune response and alleviating the hepatocellular damage in IR-stressed OLT. The experimental data for the mouse OLT study are shown (Supplemental Table 1).

### CC1-deficient OLT recipients are susceptible to NET formation in vivo.

**Figure 3:**
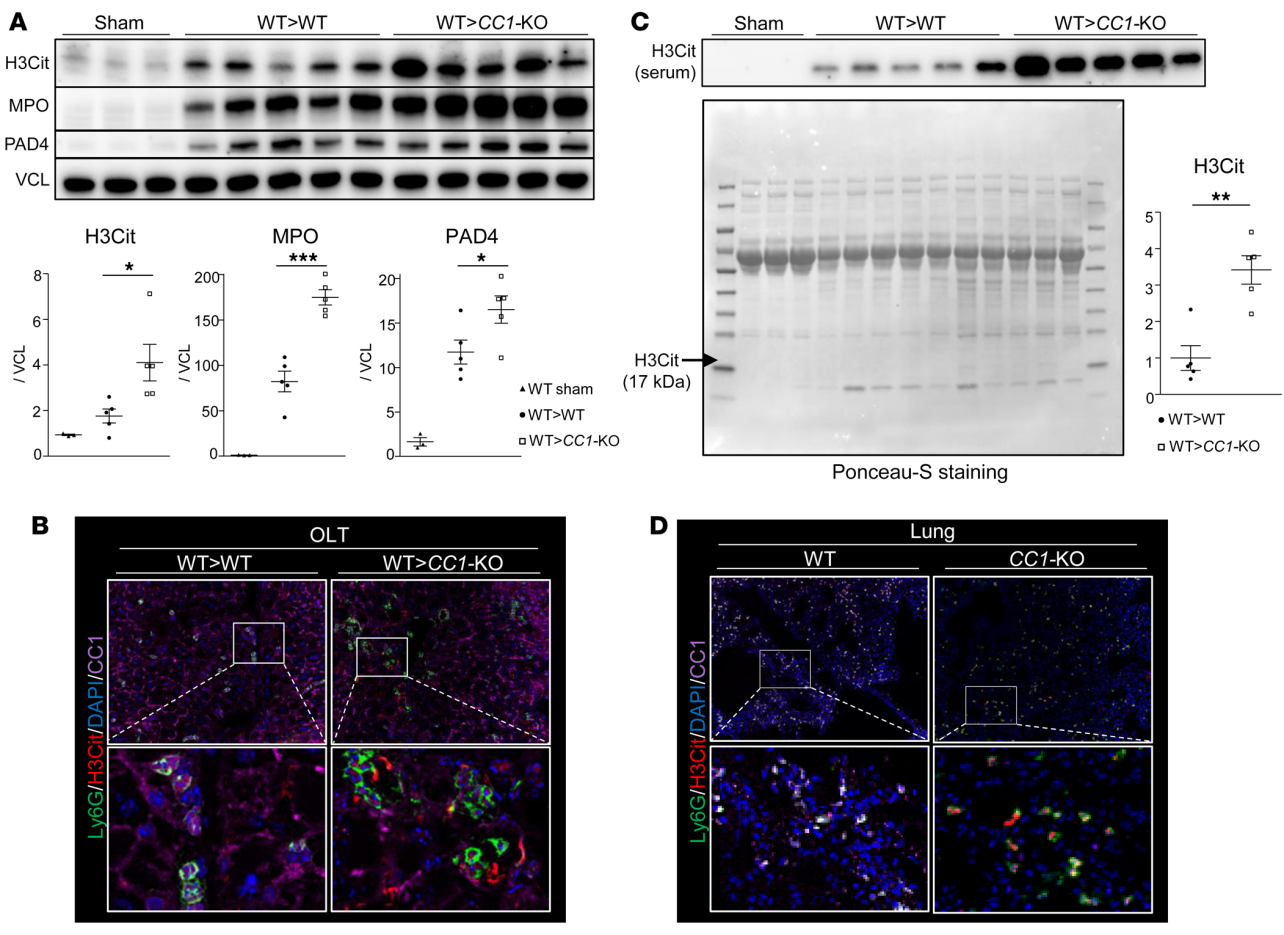
NETs in *CC1*-deficient OLT. (**A**) WB of H3Cit, MPO, and PAD4 in sham-treated and WT livers transplanted into WT versus *CC1*-KO recipients. Vinculin antibody (VCL) was used as an internal control. Data are represented as mean ± SEM. *P < 0.05; ***P < 0.001, 1-way ANOVA followed by Tukey’s HSD test. *n* = 3 (sham); *n* = 5/group (OLT). (**B**) Representative (*n* = 3) IF images of H3Cit (red), CC1 (purple), Ly6G (green), and DAPI (blue) in OLT (WT→WT or *CC1*-KO→WT) are shown. Original magnification, ×400. (**C**) WB of serum H3Cit expression and Ponceau-S staining of the PVDF membrane. The relative intensity of H3Cit expression in *CC1*-KO recipients was evaluated by comparing the averages of H3Cit expression in WT recipients’ serum. Data are represented as mean ± SEM. **P < 0.01, Student’s *t* test. *n* = 5 each (OLT). (**D**) Representative (*n* = 3) IF images of H3Cit (red), *CC1* (purple), Ly6G (green), and DAPI (blue) in the lungs of OLT recipients (WT→WT and WT→*CC1*-KO). Original magnification, ×200 (top rows); ×1200 (bottom rows).

Since NETs contribute to the pathogenesis of IRI (15–17) and NET-affiliated proteins, while CC1 significantly decreases cell adhesion, migration, and metastasis in colon cancer (11), we hypothesized that CC1 signaling might regulate NETosis in IR-stressed mouse OLT. Indeed, WB-assisted expression of NET-related markers, citrullinated histone H3 (H3Cit), myeloperoxidase (MPO), and protein arginine deiminase 4 (PAD4) were significantly enhanced in livers transplanted into *CC1*-KO compared with WT recipients (Figure 3A). In agreement with the latter, WT liver grafts in *CC1*-deficient mice (WT→*CC1*-KO) showed an increased frequency of Ly6G^+^H3Cit^+^ cells (Figure 3B), accompanied by enhanced H3Cit sera levels, compared with the WT→WT group (Figure 3C). Enhanced Ly6G^+^H3Cit levels in the lungs of *CC1*-deficient OLT recipients (Figure 3D) imply that disruption of neutrophil CC1 signaling triggered systemic NET release in distal organs in response to hepatic IR stress. Separate IF images are shown in Figure 3, C and D (Supplemental Figure 2).

### CC1-null neutrophils are susceptible to NET formation in vitro.

**Figure 4:**
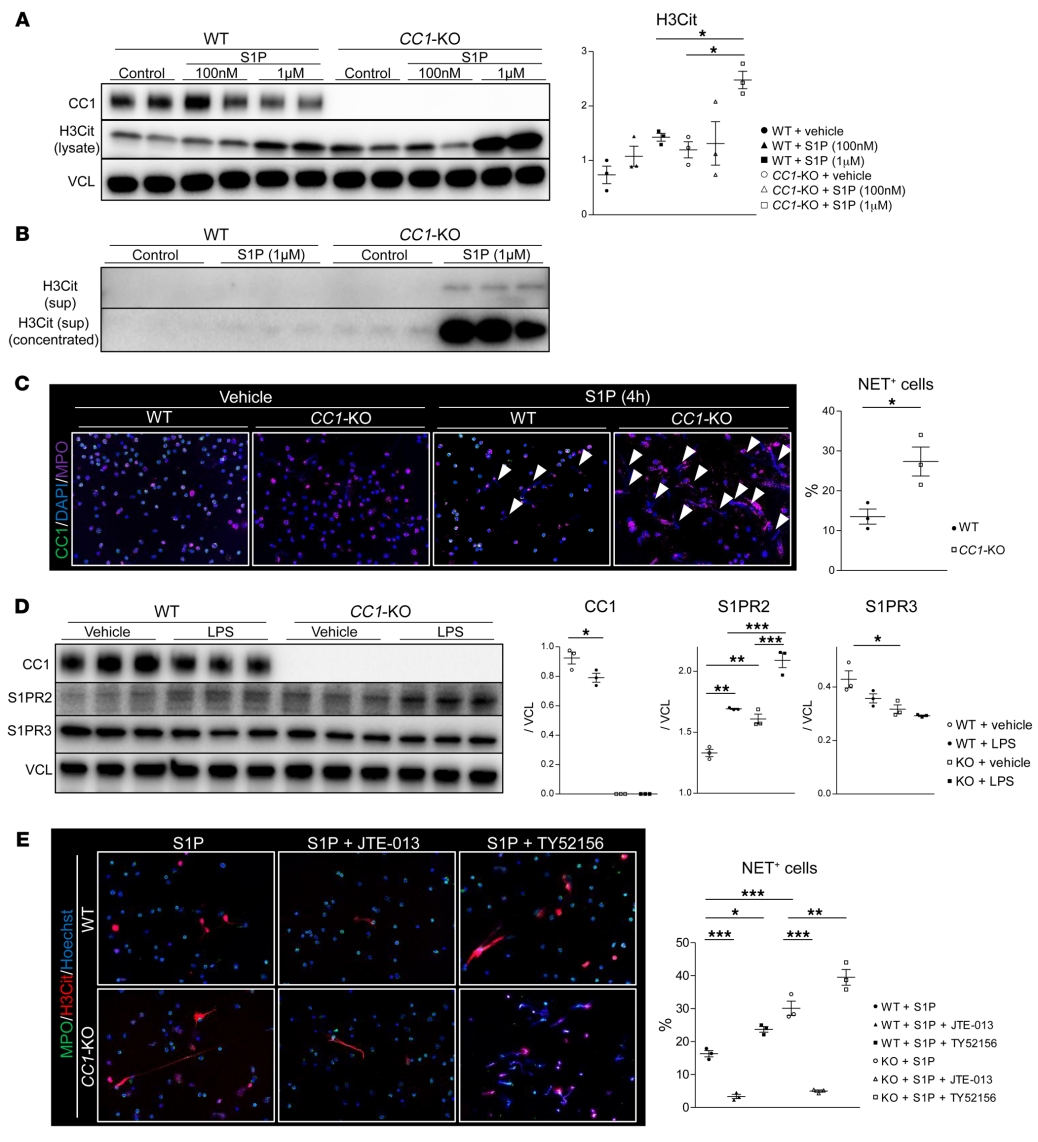
CC1 regulates NETosis via the S1PR2/S1PR3 signaling axis. (**A**) WB of CC1, H3Cit (lysate), and VCL in WT or *CC1*-KO neutrophil cultures after S1P stimulation (100 nM or 1 μM, 4 hours). VCL was used as an internal control. (**B**) WB of H3Cit in the culture media of WT or *CC1*-KO neutrophils stimulated with S1P (1 μM, 4 hours). (**C**) Representative (*n* = 3) IF images of *CC1*, MPO, and DAPI in WT versus *CC1*-null neutrophils stimulated with S1P (1 μM, 4 hours) and quantitated for NET^+^ cells. Arrowheads indicate nucleus extrusion. Data are represented as mean ± SEM. Original magnification, ×200. *P < 0.05, Student’s *t* test, *n* = 3/group. (**D**) WB-assisted detection and relative intensity ratio of *CC1*, S1PR2, and S1PR3 expression in LPS-treated WT and *CC1*-KO neutrophils (500 ng/ml, 3 hours). VCL was used as an internal control for protein analysis. Data are represented as mean ± SEM. *P < 0.05; **P < 0.01; ***P < 0.001, 1-way ANOVA followed by Tukey’s HSD test. *n* = 3/group. (**E**) Representative (*n* = 3) IF images of MPO (green), H3Cit (red), and Hoechst 33342 (blue) in WT versus *CC1*-null neutrophils stimulated with S1P (1 μM, 4 hours) in the presence of JTE-013 (10 μM, 0.5 hours) or TY52156 (10 μM, 0.5 hours) and quantification of NET^+^ cells. Original magnification, ×200. Data are represented as mean ± SEM. *P < 0.05; **P < 0.01; ***P < 0.001, 1-way ANOVA followed by Tukey’s HSD test. *n* = 3/group.

CC1 signaling was reported to regulate hepatic metabolism, while *CC1*-KO mice developed NASH in response to a high-fat diet (HFD) (14). Others have shown that human and mouse neutrophils undergo NETosis via the S1P/S1P receptor 2 (S1PR2) pathway (12). Since S1P serves as a bioactive lipid in NASH pathogenesis (18), we reasoned that *CC1*-null neutrophils might be susceptible to S1P stimulation. Indeed, after the disruption of CC1 signaling, neutrophils from *CC1*-KO mice became highly sensitive to NET formation, as evidenced by enhanced H3Cit expression in cell lysates and culture media (Figure 4, A and B). In addition, *CC1* deficiency enhanced DNA extrusion in response to S1P compared with *CC1*-proficient (WT) neutrophils (Figure 4C).

### CC1 regulates the S1PR2/S1PR3 axis and determines the sensitivity to NET formation.

To study how *CC1* deficiency enhanced susceptibility to NETosis, we screened the frequency of S1P receptors in *CC1*-proficient (WT) and *CC1*-null (KO) neutrophil cultures. As shown in Figure 4D, S1PR2 expression was significantly upregulated, while S1PR3 was suppressed in *CC1*-KO compared with WT cells. Notably, S1PR2 levels increased in WT and *CC1*-KO neutrophils after TLR4 engagement with LPS, while S1PR3 expression further decreased after LPS stimulation, accompanied by CC1 downregulation. These results indicate that CC1 negatively regulates S1PR2 while positively regulating S1PR3 signaling. Indeed, unlike enhanced S1PR2 levels in OLT-infiltrating *CC1*-null neutrophils, intrahepatic S1PR3 was relatively unchanged (Supplemental Figure 3, A and B) despite concomitantly enhanced neutrophil infiltrate (Figure 2F).

To determine how the S1PR2/S1PR3 axis may affect NET formation, neutrophils from WT and *CC1*-KO mice pretreated with S1PR2 or S1PR3 antagonist (JTE-013 and TY52156, respectively) were stimulated with S1P. As shown in Figure 4E, S1PR2 ligation attenuated, while S1PR3 ligation exacerbated, NET deployment in WT and *CC1*-KO neutrophil cultures. These results indicate that S1PR2 signaling accelerates NETs, while S1PR3 signaling acts as a counterbalance. Thus, by accelerating (via S1PR2) versus curtailing (via S1PR3) NET formation in response to S1P stimulation, *CC1* deficiency dysregulated S1PR2/S1PR3 signaling to facilitate NETosis in neutrophil cultures. Figure 4, C and E, shows separate IF images (Supplemental Figure 4)

### IR stress creates an S1P-enriched OLT environment.

**Figure 5:**
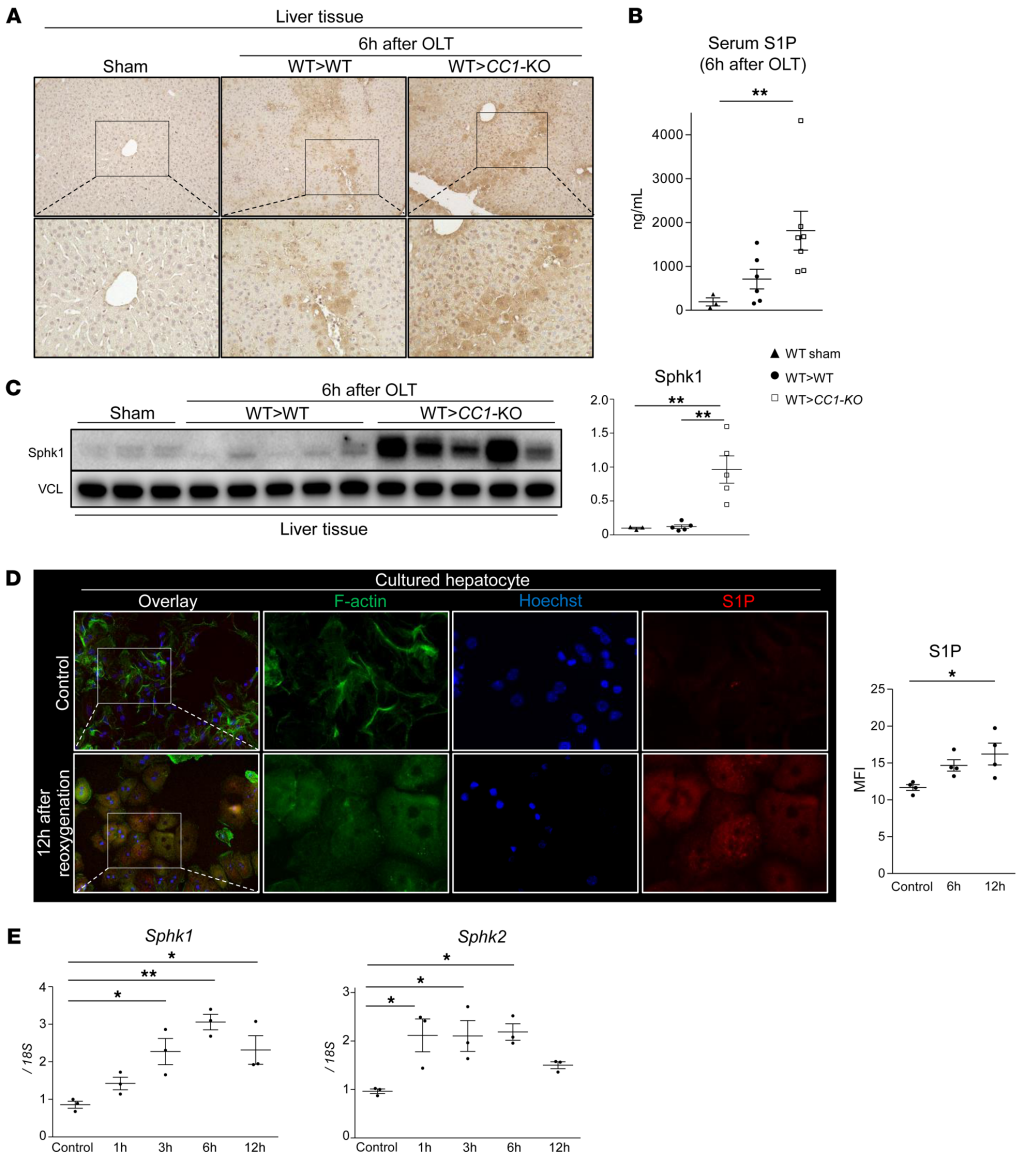
IR stress promotes an S1P-enriched OLT environment. (**A**) Representative (*n* = 2) immunohistochemistry staining for S1P in sham livers and OLT. Original magnification, ×200 (top row); ×400 (bottom row). (**B**) Serum S1P levels determined by ELISA. (**C**) WB of Sphk1 in sham-treated and OLT livers (WT→WT versus WT→*CC1*-KO). VCL was used as an internal control. (**D**) Representative (*n* = 3) IF images of F-actin (green), Hoechst 33342 (blue), and S1P (red) in reoxygenated hepatocyte cultures. Original magnification, ×400 (left row); ×1000 (right 3 rows). (**E**) Time-dependent mRNA expression pattern coding for Sphk1 and Sphk2 in cultured murine hepatocytes subjected to hypoxia reoxygenation. Data are normalized to 18S gene expression. For **B**, **C**, **D**, and **E**, data are represented as mean ± SEM. *P < 0.05; **P < 0.01, 1-way ANOVA followed by Tukey’s HSD test. *n* = 3–5/group (**B** and **C**); *n* = 3/group (**D** and **F**).

Having demonstrated the susceptibility of *CC1*-null neutrophils to NET formation in vitro, we then analyzed the expression of S1P, a ligand of S1PRs, in our mouse OLT model. Using immunohistochemistry studies of liver tissue, we identified hepatocytes as a principal source of S1P in IR-stressed OLT (Figure 5A). In addition, we showed that serum S1P levels were significantly elevated in *CC1*-KO recipients compared with sham-treated controls. However, there was no difference between sham-treated versus WT and WT versus *CC1*-KO hosts (Figure 5B). WB of sphingosine kinase 1 (Sphk1), an enzyme that catalyzes the phosphorylation of sphingosine to generate S1P, was enhanced selectively in WT livers transplanted into *CC1*-KO hosts (Figure 5C). To confirm our in vivo findings, stressed WT hepatocyte cultures subjected to 1% hypoxia, followed by reoxygenation, showed upregulated expression of S1P in parallel with increased Sphk1/2 mRNA levels (Figure 5, D and E). We conclude that IR stress enriches hepatocytes in S1P, which is then released into the host circulation.

### Neutrophil CC1 signaling regulates autophagy-driven NETosis via the S1PR2/S1PR3 axis.

Since autophagy degradation has been reported in the pathogenesis of NETosis (19), we asked whether S1P/CC1 signaling can affect the autophagy pathway. Indeed, autophagy-related proteins, Vps34, Beclin1, p62, and LC3B, all showed a time-dependent increased trend in WT and *CC1*-deficient neutrophil cultures under S1P stimulation, in parallel with enhanced H3Cit in cell lysates and culture media (Supplemental Figure 5).

**Figure 6:**
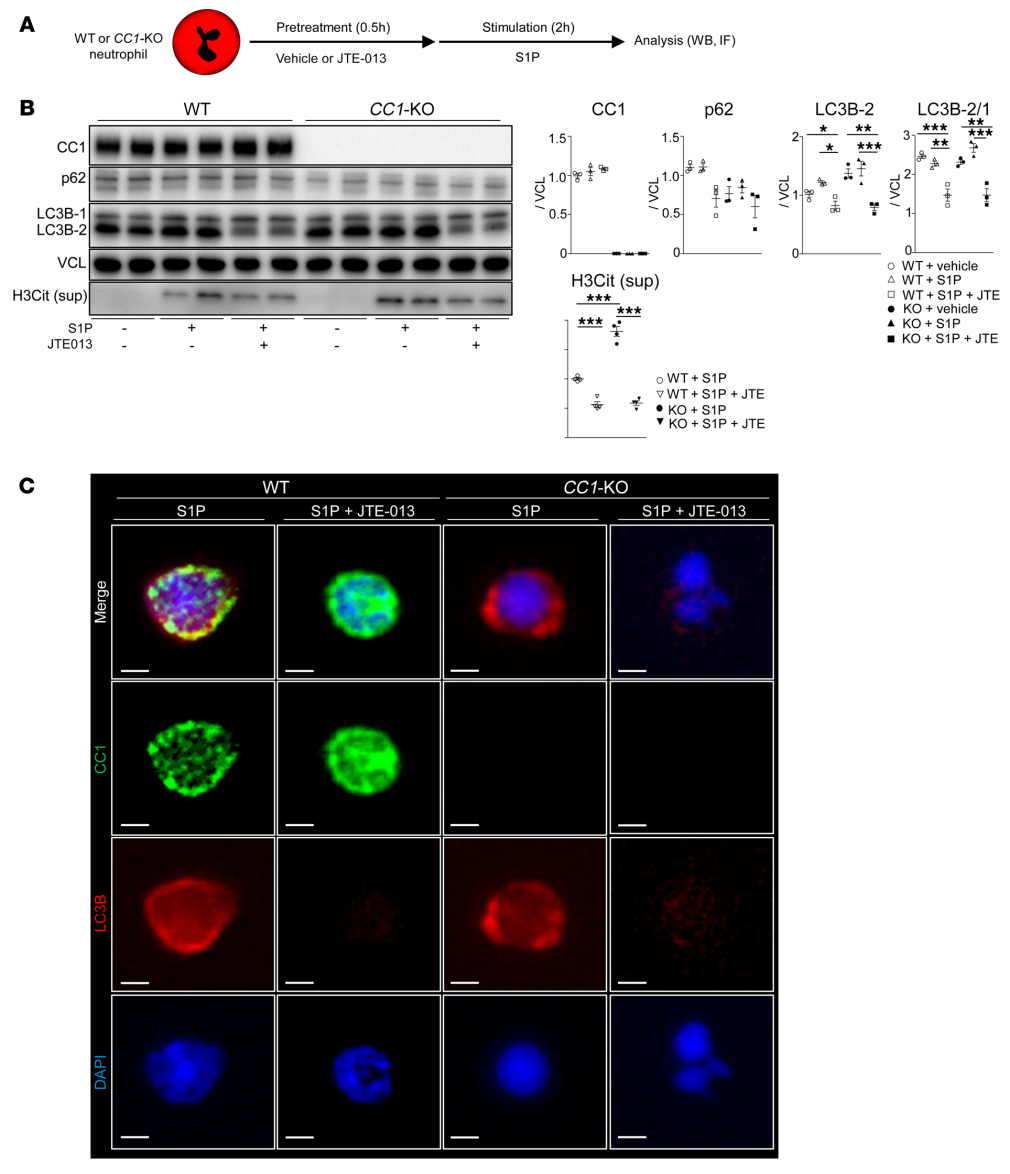
S1PR2 signaling ligation suppresses LC3B lipidation. (**A**) Schematic of cell-culture study. (**B**) WB of CC1, p62, LC3B, and H3Cit (culture media) expression in WT versus *CC1*-KO neutrophils treated with S1P (1 μM, 2 hours) with or without adjunctive JTE-013 (10 μM, 0.5 hours). Data are represented as mean ± SEM. *P < 0.05; **P < 0.01; ***P < 0.001, 1-way ANOVA followed by Tukey’s HSD test. *n* = 3/group. (**C**) Representative (*n* = 3/group) IF images of CC1 (green), LC3B (red), and DAPI (blue) in WT versus *CC1*-KO neutrophils stimulated with S1P (1 μM, 2 hours) with or without adjunctive JTE-013 (10 μM, 0.5 hours). Scale bars: 2 μm.

**Figure 7:**
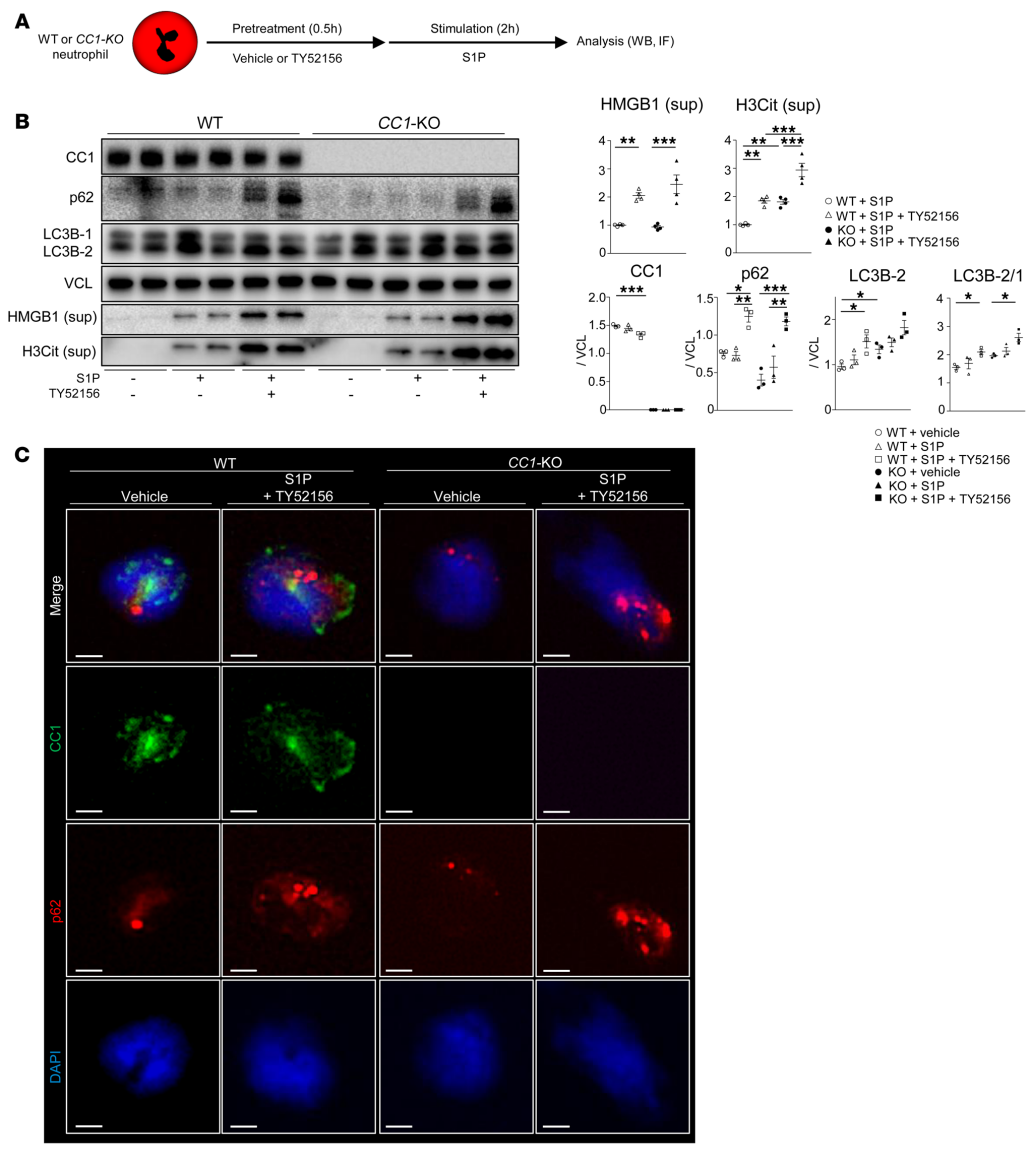
S1PR3 signaling disruption augments p62 and LC3B expression. (**A**) Schematic of cell-culture study. (**B**) WB of CC1, p62, LC3B, HMGB1, and H3Cit in WT versus *CC1*-KO neutrophils stimulated with S1P (1 μM, 2 hours) with or without adjunctive TY52156 (10 μM, 0.5 hours). VCL was used as an internal control. Data are represented as mean ± SEM. *P < 0.05; **P < 0.01; ***P < 0.001, 1-way ANOVA followed by Tukey’s HSD test. *n* = 3/group. (**C**) Representative (*n* = 3/group) IF images of CC1 (green), p62 (red), and DAPI (blue) in WT versus *CC1*-KO neutrophil cultures stimulated with S1P (1 μM, 2 hours) with TY52156 (10 μM, 0.5 hours) pretreatment. Scale bars: 2 μm.

We then sought to investigate how the S1PR2/S1PR3 axis may regulate neutrophil autophagy and NET formation in our neutrophil in vitro model. First, WT and *CC1*-null neutrophils were stimulated with S1P with or without the S1PR2 antagonist (Figure 6A). Indeed, LC3B-2 lipidation and H3Cit expression were significantly suppressed by adjunctive S1PR2 ligation, concomitant with decreased p62 (Figure 6B). Representative IF stains shown in Figure 6C identified strong downregulation of LC3B after disrupting S1PR2 signaling in WT and *CC1*-deficient neutrophil cultures. Next, we analyzed the impact of S1PR3 signaling on autophagy and NET deployment (Figure 7A). Figure 7B shows enhanced LC3B lipidation in WT and *CC1*-KO neutrophils with simultaneous accumulation of p62 after S1PR3 ligation. The release of H3Cit and HMGB1 into culture media significantly increased in WT and *CC1*-KO neutrophils under S1PR3 ligation compared with S1P stimulation alone. In addition, inhibition of S1RP3 signaling further exacerbated H3Cit expression in *CC1*-deficient rather than WT cells. The basal p62 levels in *CC1*-KO neutrophils were lower than in WT counterparts under normal steady-state conditions (Figure 6B and Figure 7B). After S1P plus TY52156 treatment, p62 levels became comparable in *CC1*-KO and *CC1*-proficient (WT) cultures, suggesting that neutrophil autophagy was more profoundly impaired after the disruption of *CC1* signaling. Figure 7C confirms a massive accumulation of p62 after S1PR3 inhibition in WT and *CC1*-KO neutrophil cultures. Thus, we conclude that S1PR2 signaling counteracts while S1PR3 promotes autophagy induction in *CC1*-deficient neutrophil cultures.

### The late stage of autophagy/lysosomal quality promotes H3Cit generation.

**Figure 8:**
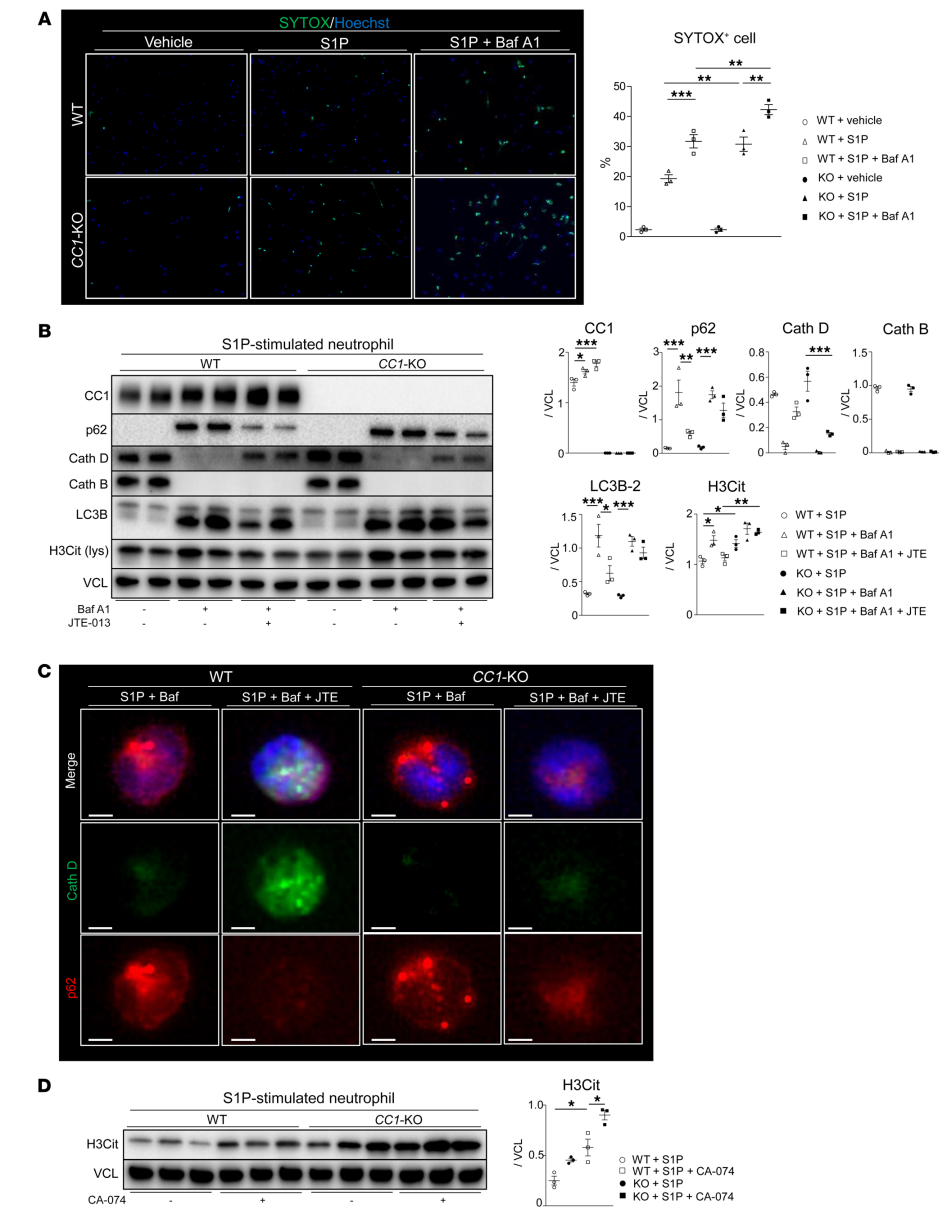
Late autophagy stage is essential for H3Cit expression. (**A**) Representative IF images of SYTOX green (green) and Hoechst 33342 (blue) in WT or *CC1*-KO neutrophils conditioned with a vehicle, S1P, and S1P (1 μM, 4 hours) plus Baf A1 (100 nM, 0.5 hours) pretreatment, and quantification of SYTOX green–positive cells. Data are represented as mean ± SEM. **P < 0.01; ***P < 0.001, 1-way ANOVA followed by Tukey’s HSD test. *n* = 3/group. Original magnification, ×200. (**B**) WB of CC1, p62, CathD, CathB, LC3B, and H3Cit (cell lysates) in WT versus *CC1*-KO neutrophils stimulated with *S1P* (1 μM, 4 hours) with Baf A1 (100 nM, 0.5 hours) alone or Baf A1 plus JTE-013 (100 nM/10 μM, 0.5 hours). VCL was used as an internal control. Data are represented as mean ± SEM. *P < 0.05; **P < 0.01; ***P < 0.001, 1-way ANOVA followed by Tukey’s HSD test. *n* = 4/group. (**C**) Representative (*n* = 3/group) IF images of CathD (green), p62 (red), *CC1* (purple), and DAPI (blue) in S1P-stimulated (1 μM, 4 hours) WT or *CC1*-KO neutrophils with Baf A1 (100 nM, 0.5 hours) or Baf A1 plus JTE-013 (100 nM/10 μM, 0.5 hours) pretreatment. Scale bars: 2 μm. (**D**) WB of H3Cit in WT versus *CC1*-KO neutrophils stimulated with S1P (1 μM, 4 hours) with or without CA-074 pretreatment (10 μM, 0.5 hours). VCL was used as an internal control. Data are represented as mean ± SEM. *P < 0.05, 1-way ANOVA followed by Tukey’s HSD test. *n* = 3/group.

After uncovering the regulatory function of the S1PR2*/*S1PR3 axis in the autophagy pathway, we next sought to investigate the impact of autophagy on S1P-induced NET formation (Supplemental Figure 6). WT and *CC1*-null neutrophil cultures were pretreated with bafilomycin A1 (Baf A1), a selective vacuolar H^+^-ATPase inhibitor, which suppresses autophagosome-lysosome fusion, followed by S1P stimulation. As shown in Figure 8A, WT and *CC1*-KO neutrophils pretreated with Baf A1 exhibited enhanced NET formation compared with S1P stimulation alone, indicating that autophagy is essential for preventing S1P-induced NETosis. To determine whether S1PR2 ligation may attenuate NET deployment in the autophagy-deficient environment, neutrophils were pretreated with Baf A1 (autophagy inhibitor), followed by JTE-013 (S1PR2 antagonist) and S1P stimulation. As shown in Figure 8B, H3Cit expression was enhanced in adjunctively Baf A1–conditioned compared with S1P-only stimulated cells in concert with the increase of p62 and LC3B lipidation. We observed that Baf A1 treatment depleted lysosomal cysteine proteases, cathepsin B (CathB) and CathD, while adjunctive S1PR2 ligation more efficiently restored CathD in WT than *CC1*-KO neutrophils. Additionally, S1PR2 ligation in WT neutrophils suppressed H3Cit and p62 in the presence of Baf A1, concomitant with CathD restoration. As these effects were dampened in *CC1*-null neutrophil cultures, we conclude that CC1 signaling regulates S1P-stimulated lysosomal stability. When IF images were analyzed, we observed that S1PR2 ligation reinstated CathD and decreased p62 in WT cultures (Figure 8C). In contrast, although S1PR2 ligation failed to restore CathB in S1P-stimulated WT and *CC1*-KO neutrophils, CathB inhibition accelerated S1P-induced NET formation in WT and *CC1*-KO neutrophils (Figure 8D), suggesting that CathD and CathB are essential in S1P-triggered NETosis. Figure 8, A and C, shows separate IF images (Supplemental Figure 7).

Next, to investigate the impact of the early autophagy phase on NETs, we pretreated *CC1*-deficient neutrophils with wortmannin, a nonspecific, covalent inhibitor of phosphoinositide 3 kinases, to prevent the initiation/elongation stage of autophagy, followed by S1P stimulation. Consistent with the Baf A1 treatment data, autophagy inhibition markedly exacerbated NETosis (Supplemental Figure 8). However, in contrast to the Baf A1 regimen, adjunctive S1PR2 ligation attenuated cit H3 levels and HMGB1 release in a wortmannin-conditioned *CC1*-deficient environment. Our findings are consistent with the idea that the late stage of autophagy and lysosomal function is essential for NET formation, as determined by distinctive S1PR2 and S1PR3 signaling in WT and *CC1*-KO neutrophil cultures.

### Reconstitution of PMN^DTR^ transgenic mice with CC1-null neutrophils exacerbates hepatic IRI.

**Figure 9:**
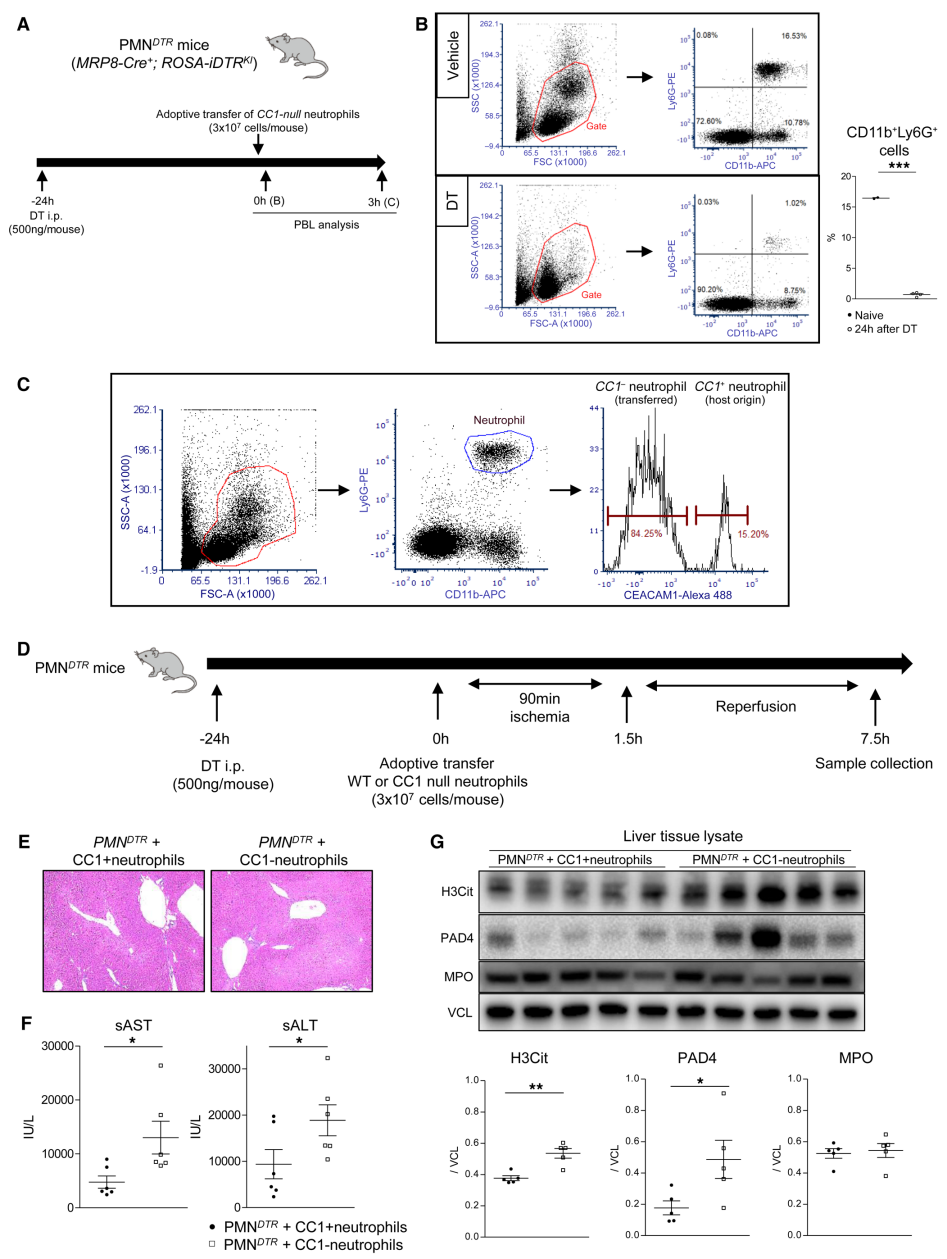
Adoptive transfer of *CC1*-null neutrophils exacerbates liver IRI and enhances H3Cit expression in PMN^DTR^ mice. (**A**) Experimental schematic of DT treatment, followed by adoptive transfer of *CC1*-null neutrophils into PMN^DTR^ mice. (**B**) Gating peripheral blood analysis with CD11b^+^ and Ly6G^+^ cells by flow cytometry and the proportion of CD11b^+^Ly6G^+^ cells after vehicle versus DT administration. Data are represented as mean ± SEM. ***P < 0.001, Student’s *t* test. *n* = 2–3/group. (**C**) Analysis of CC1^+^ and CC1^–^ neutrophil populations in PMN^DTR^ mouse conditioned with *CC1*-null neutrophils. (**D**) Experimental schematic of PMN^DTR^ treatment, followed by neutrophil reconstitution and warm hepatic IRI. (**E**) Representative H&E staining of IR-stressed livers. Original magnification, ×100. (**F**) sAST and sALT levels. Data are represented as mean ± SEM. *P < 0.05, Student’s *t* test. *n* = 5/group. (**G**) WB of H3Cit, PAD4, and MPO in post-IRI livers in PMN^DTR^ mice. VCL was used as an internal control. Data are represented as mean ± SEM. *P < 0.05; **P < 0.01, Student’s *t* test. *n* = 5/group.

Even though *CC1*-KO mouse recipients were highly susceptible to NET formation in the IRI-OLT model, the *CC1* signaling defect could affect multiple nonparenchymal cells, such as circulating neutrophils, T cells, macrophages, and dendritic cells. To specifically focus on neutrophil CC1 function, we generated a diphtheria toxin–inducible (DT-inducible) neutrophil depletion transgenic mouse model, as reported (20) and shown in Figure 9A. After DT treatment abolished the peripheral CD11b^+^Ly6G^+^ cell population (Figure 9B), adoptive transfer of *CC1*-null neutrophils recreated the *CC1*-deficient neutrophil population in PMN*^DTR^* mouse recipients (Figure 9C). Figure 9D depicts the schematic for neutrophil reconstitution (3 × 10^7^ cells i.v.), followed by warm hepatic IR insult, in a DT-inducible PMN*^DTR^* test model. Consistent with WT mouse IRI-OLT data, reconstitution of PMN*^DTR^* mice with *CC1*-deficient neutrophils significantly increased hepatic IRI and NETosis compared with PMN*^DTR^* mice conditioned with *CC1*-proficient (WT) cells. This was evident by increased hepatic sinusoidal congestion, edema vacuolization, and necrosis by histology (Figure 9E), a higher serum transaminase release (Figure 9F), and significantly enhanced hepatic PAD4 and H3Cit expression patterns (Figure 9G).

As Ly6G expression in response to S1P stimulation remained unchanged in WT and *CC1*-KO neutrophil cultures (Supplemental Figure 9A), we thought hepatic Ly6G levels might reflect the number of liver-infiltrating neutrophils. Indeed, WB-assisted hepatic Ly6G expression and the frequency of Ly6G^+^ cells infiltrating post-IR livers were comparable (Supplemental Figure 9, B and C). These results suggest that *CC1*-null neutrophils readily undergo NETosis, while enhanced NET formation in PMN*^DTR^* mice repopulated with *CC1*-KO neutrophils was not due to increased hepatic neutrophil infiltration. In addition, NET-targeted CI-amidine pretreatment canceled differences in IR-triggered hepatocellular damage and hepatic/serum H3Cit levels, seen otherwise in PMN*^DTR^* mice repopulated with WT versus *CC1*-KO neutrophils (Supplemental Figure 10, A–D). Thus, liver IRI in PMN*^DTR^* mice repopulated with *CC1*-null neutrophils was mostly NET dependent. Of note, PMN*^DTR^* mice conditioned with *CC1*-deficient neutrophils showed a trend toward higher (albeit not significant) serum transaminase release because of putative proinflammatory phenotype. Indeed, in agreement with what was found by others (21), *CC1*-null neutrophils showed higher expression of TNFA and IL1B in response to LPS (Supplemental Figure 10E). These findings from the PMN*^DTR^* mouse system are consistent with the regulatory function of neutrophil *CC1* signaling in IR-induced hepatocellular damage and NETosis.

### CC1-L/CathG ratio negatively correlates with the hepatocellular function in human OLT.

**Figure 10:**
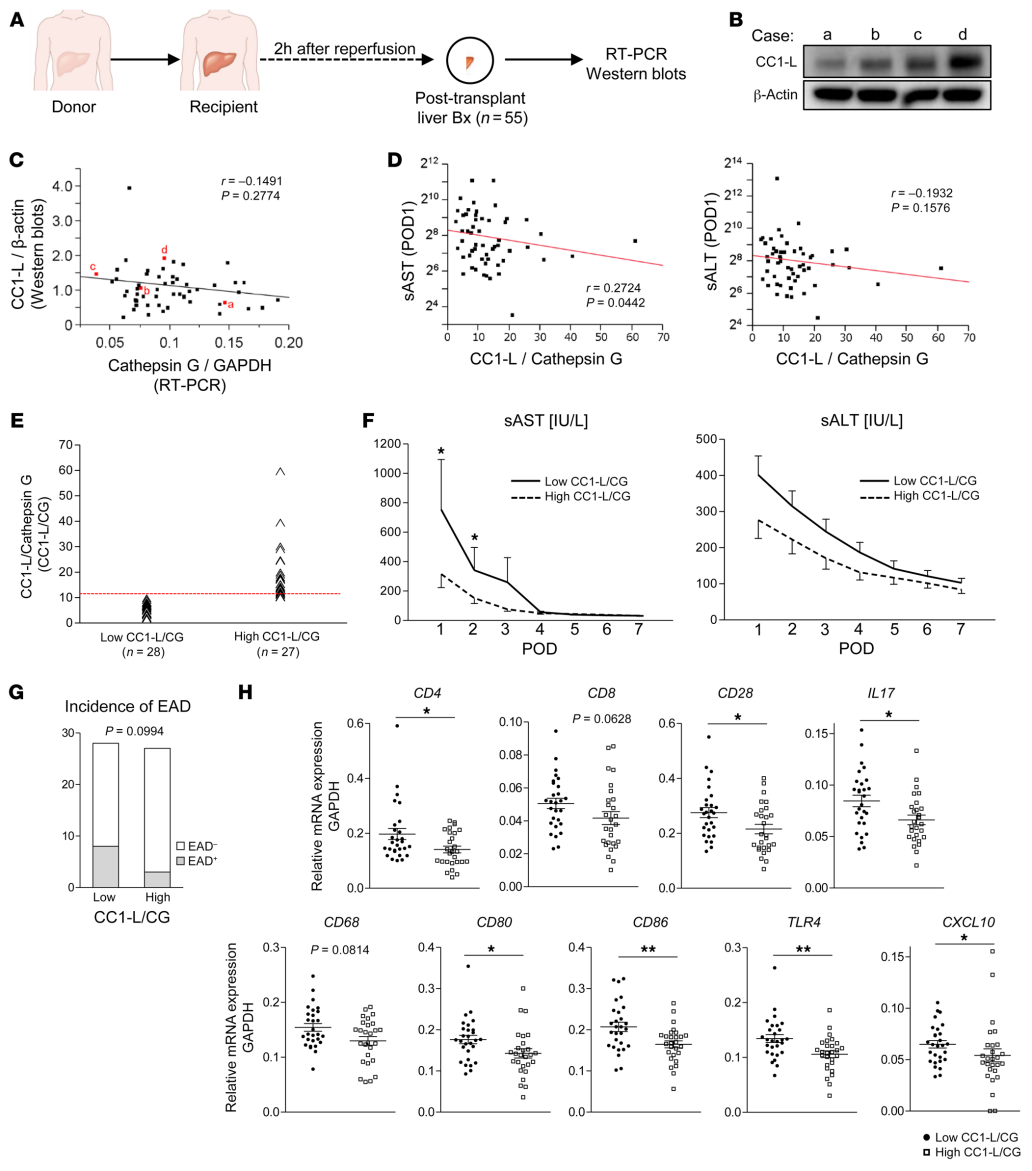
CC1-L level and CC1-L/*CathG* ratio are associated with hepatocellular function and innate/adaptive immune responses in OLT patients. (**A**) Human OLT Bx (*n* = 55), collected 2 hours after reperfusion, were analyzed for CC1-L by WB with β-actin normalization and for CathG by qRT-PCR with normalization to GAPDH. (**B**) Four representative WB of CC1-L expression are shown. Case a, low–CC1-L; cases b/c, intermediate CC1-L; case d, high–CC1-L. (**C**) Relationship between CC1-L and CathG. (**D**) Relationship between CC1L/CathG and sAST/sALT at POD1. *r*, Spearman’s correlation coefficient. (**E**) Human OLT Bx samples (2 hours after reperfusion) were classified into low (*n* = 28) and high (*n* = 27) CC1-L/CathG groups. (**F**) sAST and sALT levels at POD1–7. *P < 0.05, Mann-Whitney *U* test. Data are represented as mean ± SEM. (**G**) Incidence of EAD. Fisher’s exact test. (**H**) qRT-PCR–assisted detection of mRNA coding for CD4, CD8, CD28, IL17, CD68, CD80, CD86, TLR4, and CXCL-10. Data normalized to GAPDH gene expression are shown in dot plots, and bars indicate mean ± SEM. *P < 0.05; **P < 0.01, Mann-Whitney *U* test.

Having demonstrated the regulatory function of neutrophil CC1-L isoform in mice, we next aimed to validate its relevance in OLT patients. We retrospectively analyzed 55 human liver biopsies (Bx) collected at 2 hours after reperfusion. We screened them for CC1-L, with β-actin normalization (WB), and CathG, with GAPDH normalization (reverse-transcriptase PCR [RT-PCR]) (Figure 10, A and B). Hepatic CC1-L levels correlated negatively with CathG (*r* = –0.1491, *P* = 0.2774; Figure 10C), suggesting CC1-L–enriched neutrophils mitigate the proinflammatory phenotype. To test this hypothesis, we analyzed the “strength” of neutrophil CC1-L expression as a function of CathG expression levels (CC1-L/CathG) (Figure 10C). Case a shows higher CathG expression and a lower CC1-L level, while case d had lower CathG expression but a higher CC1-L level. Using this relationship ratio, we observed that the CC1-L/CathG ratio negatively correlated with early hepatocellular graft function, assessed by sAST (*r* = –0.2724, *P* = 0.0442) and sALT (*r* = –0.1932, *P* = 0.1576) at postoperative day 1 (POD1) (Figure 10D).

### CC1-L/CathG ratio determines the hepatocellular function and regulates innate/adaptive immune phenotype in human OLT recipients.

Since disruption of neutrophil CC1 signaling was critical for the murine IRI-OLT phenotype, we next aimed to evaluate the correlation between CC1-L/CathG and OLT damage in humans. Based on WB-assisted CC1-L and CathG expression patterns, posttransplant Bx samples collected from 55 OLT patients were divided into low–CC1-L/CathG (*n* = 28) and high–CC1-L/CathG (*n* = 27) groups according to the median split (Figure 10E). Patients’ demographic and clinical data are shown in Supplemental Tables 2 and 3. There was no correlation between CC1-L levels and the recipient/surgical parameters, including age, sex, race, BMI, disease etiology, ABO compatibility, pretransplant blood tests, preoperative hospital stay, cold/warm ischemia time, or blood transfusions. The model for end-stage liver disease (MELD) score, a measure of how severe a patient’s liver disease is, and prothrombin time/international normalized ratio (PT/INR) were elevated in the high–CC1-L/CathG group (*P* = 0.006 and 0.013, respectively) (Supplemental Table 2). Except for donor age, which was higher in the high–CC1-L/CathG group (*P* = 0.015), there was no correlation between CC1-L grouping and other donor data (Supplemental Table 3), including sex, race, BMI, preprocurement blood tests, and donation status (after circulatory or brain death).

The low–CC1-L/CathG group had significantly higher sAST at POD1–2 (Figure 10F) and trended toward higher sALT levels, reflecting a deteriorated OLT function. Despite the difference failing to reach statistical significance, low–CC1-L/CathG cases showed an increased frequency of early allograft dysfunction (EAD) compared with the high–CC1-L/CathG group (Figure 10G, 28.6% versus 11.1%).

We then analyzed innate and adaptive gene programs in human liver Bx obtained at 2 hours after OLT. Consistently, low–CC1-L/CathG grafts exhibited increased mRNA level coding for T cell activation markers CD4 (*P* = 0.0246), CD8 (*P* = 0.0628), CD28 (*P* = 0.0216), and IL-17 (*P* = 0.0216) as well as macrophage activation markers CD68 (*P* = 0.0814), CD80 (*P* = 0.0188), CD86 (*P* = 0.0072), TLR4 (*P* = 0.0039), and CXCL10 (*P* = 0.0257) (Figure 10H). Hence, the low–CC1-L/CathG intragraft ratio was accompanied by accelerated postreperfusion innate/adaptive immune activation and enhanced hepatocellular damage in the early post-OLT phase.

To assess the contribution of CC1 signaling in human liver IRI, we next analyzed CC1-L levels in pretransplant liver Bx by WB. The post-/pretransplant ratios were calculated to determine the CC1-L profile (ΔCC1-L). The peritransplant CC1-L/CathG (ΔCC1-L/CG) ratio and clinical parameters were examined, and results are shown in Figure 10. Indeed, the ΔCC1-L/CG ratio was negatively correlated with posttransplant transaminase release at POD1 and H3Cit levels at 2 hours after reperfusion. When ΔCC1-L/CG groups were assessed by the median split, the low–ΔCC1-L/CG patient cohort showed enhanced hepatocellular damage, increased EAD incidence, and augmented proinflammatory gene profiles (Supplemental Figure 11). These findings align with our hypothesis that CC1-L contributed to NET formation and clinical outcomes in liver transplant patients.

### Plasma H3Cit levels determine the hepatocellular function in human OLT.

**Figure 11:**
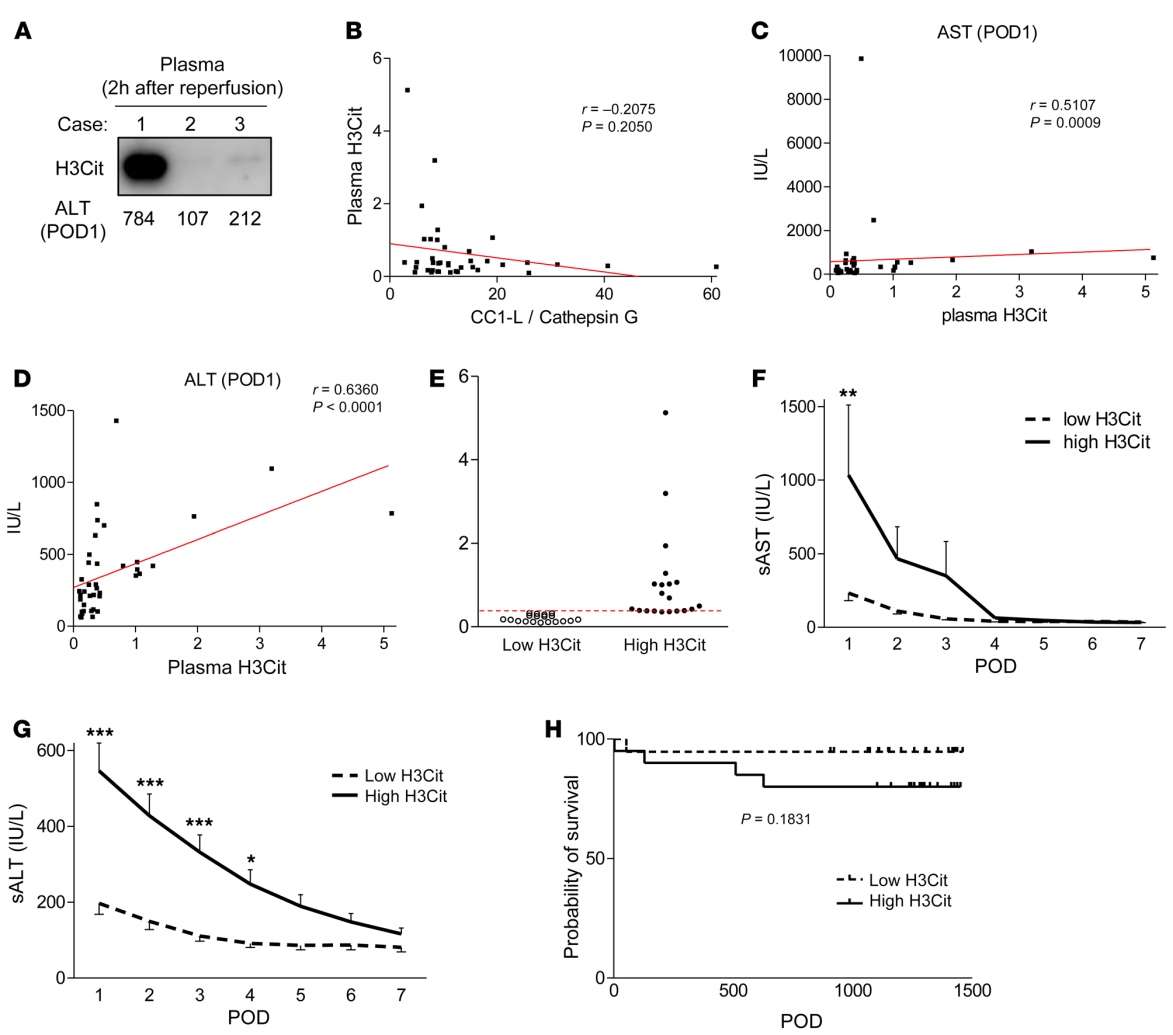
Post-OLT H3Cit plasma levels determine the hepatocellular function in OLT patients. (**A**) WB of H3Cit in human plasma samples collected 2 hours after OLT and sALT levels at POD1. Case 1, high H3Cit; case 2, low H3Cit; case 3, intermediate H3Cit. (**B**) Relationship between plasma H3Cit and CC1L/CathG ratio. (**C** and **D**) Relationship between plasma H3Cit and sAST/sALT at POD1. *r*, Spearman’s correlation coefficient. (**E**) Human plasma samples (2 hours after OLT) were classified into low (*n* = 19) and high (*n* = 20) H3Cit groups. (**F** and **G**) sAST and sALT levels at POD1–7. Data are represented as mean ± SEM. *P < 0.05; **P < 0.01; ***P < 0.001, Mann-Whitney *U* test. (**H**) Cumulative probability of overall graft survival. Solid line indicates high H3Cit, while dotted line depicts low H3Cit in OLT patients. Kaplan-Meier method, log-rank test.

Figure 11A shows representative WB-assisted detection of H3Cit in human blood collected at 2 hours after OLT (case 1, high H3Cit/sALT; case 2, low H3Cit/sALT; and case 3, intermediate H3Cit/sALT). These correlated negatively with the CC1-L/CathG ratio (*r* = –0.2075, *P* = 0.2050; Figure 11B) while showing a highly significant positive correlation with sAST (*r* = 0.5107, *P* = 0.0009; Figure 11C) and sALT (*r* = 0.6360, *P* < 0.0001; Figure 11D) at POD1. We then divided plasma samples from 39 OLT patients into low-H3Cit (*n* = 19) and high-H3Cit (*n* = 20) groups by the median split (Figure 11E). Indeed, high-H3Cit individuals had significantly higher levels of sAST at POD1 (Figure 11F) and sALT at POD1–4 (Figure 11G) and an inferior trend for graft survival (Figure 11H). These results highlight the protective function of neutrophil CC1-L against liver damage and NETosis in OLT patients.

Next, we investigated the relationship between circulating H3Cit levels and peripheral blood profiles in OLT patients. Since our center does not routinely measure neutrophil frequencies, we had access to peripheral blood neutrophil data from 27 patients (of 39 individuals with known WBC counts). First, we did not find any correlation between H3Cit levels and WBC or platelet counts at pretransplant, POD0 (day of transplantation), and POD1 time points (Supplemental Figure 12, A and B). Plasma H3Cit levels negatively correlated with ΔCC1-L (Supplemental Figure 12C) while positively correlating with MPO and neutrophil elastase (NE) concentrations (Supplemental Figure 12, D and E). When divided by the median split, according to the H3Cit expression levels, the “high” H3Cit group showed enhanced hepatic mRNA levels coding for IL-17A (*P* = 0.0821), CXCL10 (*P* < 0.05), and CathG (*P* = 0.064) (Supplemental Figure 11F). When our patient cohort was analyzed for neutrophil profile, plasma H3Cit levels correlated negatively, albeit not significantly, with ΔCC1-L/CathG ratios, but positively with MPO/NE levels and transaminase release at POD1 (Supplemental Figure 13). In contrast, total WBC/neutrophil counts and neutrophil and platelet counts failed to correlate with plasma H3Cit levels. Despite small patient cohort and several factors potentially affecting peripheral blood lymphocyte (PBL) counts (e.g., blood loss, blood transfusion, etc.), these results suggest that elevated plasma H3Cit levels due to decreased neutrophil CC1-L expression in the stressed liver rather than increased neutrophil frequency per se contributed to enhanced NET formation in liver transplant patients.

## Discussion

This translational study uncovered the regulatory function of recipient-derived neutrophil CC1-L isoform in IR-stressed OLT in mice and humans. In the experimental arm, the host neutrophil *CC1*-null mutation caused hepatocellular function to deteriorate and augmented innate immune activation in mouse OLT, evidenced by histology, frequency of TUNEL^+^ cells, and the proinflammatory phenotype (Figure 2). Enhanced OLT damage was accompanied by increased levels of local (hepatic) and circulating (plasma) H3Cit, a central player in releasing nuclear chromatin, known as NETs (Figure 3). In the clinical arm of 55 patients, low–CC1-L/CathG neutrophil infiltrate worsened OLT function, enhanced innate/adaptive immune activation, and trended toward a higher incidence of EAD than in high–CC1-L/CathG phenotype individuals (Figure 10). Notably, circulating H3Cit levels showed a significant positive correlation with the hepatocellular function in OLT patients. Thus, while neutrophils have been recognized as a principal villain in tissue injury, our results show their CC1-L isoform serves as a negative checkpoint regulator of IR stress and NETosis in human and murine IRI-OLT.

Despite recipient-derived neutrophils, macrophages, and T cells readily migrating into the liver graft in the reperfusion phase, the neutrophil infiltrate indicates the severity of hepatic IRI (1). NETosis, a lytic cell death distinct from necrosis or apoptosis, is characterized by the release of decondensed chromatins, such as NE, MPO (22), and H3Cit, into the extracellular space (23), which then acts as DAMPs to accelerate sterile inflammation (24). Given the ability of NETs in cancer metastasis (8), local hepatic IR stress/inflammation triggered NETosis in peripheral blood and distal organs of OLT recipients. Indeed, the H3Cit level in peripheral blood, a reliable biomarker for NET formation, is a prognostic factor in several disease states, including asthma, sepsis, and cancer (25–27).

The question arises of how CC1 regulates NETosis in the mechanism of IR-triggered OLT damage. Consistent with a finding that neutrophil CC1 was dispensable in PMA-induced NETosis (11), LPS stimulation failed to induce NET formation in WT and *CC1*-null neutrophil cultures (our unpublished data). Even though several molecular pathways may contribute to NET pathogenesis (28), we found *CC1*-deficient neutrophils were highly sensitive to NET formation under S1P challenge, as manifested by increased H3Cit levels in cell lysate and culture media screens (Figure 4). S1P, one of the sphingolipid metabolites generated from ceramide by Sphk1 and Sphk2 (29), has been implicated in inflammation, neural communication, cancer, and metabolic diseases (e.g., diabetes, nonalcoholic fatty liver disease [NAFLD]) as well as organ IRI (18, 30). In our mouse OLT model, serum S1P levels significantly increased in *CC1*-deficient recipients (Figure 5), suggesting neutrophils were exposed to higher S1P concentrations than under steady-state *CC1*-proficient (WT) counterparts. OLT histology and cell cultures identified hepatocyte as the principal source of S1P. Indeed, hypoxia accelerated Sphk activity via HIF-1A to generate S1P in the hepatocellular carcinoma cell line (31). Once released into the extracellular space, S1P acts as a chemoattractant to recruit immune cells to inflamed tissue (32, 33). Sphk1 and S1P expression served as liver-damage markers, increasing with disease progression in a bile duct ligation model (33) and patients with chronic liver injury undergoing OLT (34). In agreement with these reports, significantly higher hepatic Sphk1 and plasma S1P aggravated liver IRI in *CC1*-KO mice. Thus, *CC1*-deficient neutrophils exposed to S1P were increasingly prone to NETosis, resulting in accelerated OLT damage. However, targeting S1P may not be therapeutically desirable, since it is indispensable for liver regeneration/sinusoidal endothelial cell integrity after liver resection (35, 36).

To the best of our knowledge, this is the first report documenting the role of the neutrophil S1PR2/3 axis in organ transplantation. Others have shown that treatment with FTY720 (fingolimod) targeting S1PR1 prevented T cell egress from lymph nodes in cardiac, kidney, and hematopoietic cell transplant models (37–39). In addition, the function of S1PRs may be cell type specific. Even though we have demonstrated that neutrophil S1PR3 ligation accelerated NETosis, macrophage S1PR3 signaling promoted proinflammatory responses against LPS challenge (40), while disruption of S1PR3 on dendritic cells attenuated allogenic response by expanding regulatory T cells (41). By regulating the S1PR2/S1PR3 axis, neutrophil CC1 determined the sensitivity to NETosis in IR-stressed OLT (Figure 4, D and E). Indeed, S1PR2 ligation attenuated NETs in S1P-stimulated WT and *CC1*-deficient neutrophil cultures in an S1PR2-dependent manner, while S1PR3 ligation exacerbated NETs. The question arises of how S1PR2/3 may regulate NETs despite S1PR2 and S1PR3 sharing a common signaling pathway (42). For example, coordinated S1PR2/3 signaling shifted macrophage activation toward a proinflammatory phenotype through G(α)i/o/PI3K/JNK (43), while Akt was implicated in LPS-induced NETosis (44). Selective S1PR2 engagement enhanced ERK1/2 in *CC1*-deficient neutrophils, while S1PR3 stimulation upregulated p38 in WT neutrophils (Hirao, unpublished observations).

Though ROS and autophagy were required for NET formation (45), ROS generation was comparable in S1P-stimulated WT and *CC1*-KO neutrophil cultures (Hirao, unpublished observations). We hypothesized that dysregulation of autophagy due to the S1PR2/3 axis imbalance could be why *CC1*-deficient neutrophils were more sensitive to S1P stimulation than their WT counterparts. Of note, S1P-S1PR2 ligation downregulated LC3B2, while S1PR3 ligation enhanced LC3B2 in parallel with increased p62 and H3Cit expression (Figure 6B and Figure 7B). These results unveiled a previously unappreciated ability of neutrophil CC1 to regulate autophagy via the S1PR2/S1PR3 axis. The mechanism by which autophagy may affect different cell types remains controversial (46, 47). We utilized pharmacological inhibitors to investigate the impact of S1PR2/S1PR3 signaling on autophagy, with Baf A1–pretreated S1P-stimulated neutrophils exhibiting enhanced LC3B-2/p62 expression and aggravated NET formation. This finding was consistent with the neutrophil protein expression pattern after disrupting S1PR3 signaling (TY52156) (Figure 7B and Figure 8B). Thus, enhanced NETosis in *CC1*-null neutrophils was due to the impaired autophagy pathway.

Dysfunction of the autophagy pathway, a finely tuned dynamic intracellular degradation process, has been implicated in various pathology states, including organ IRI (48). It remains controversial whether autophagy promotes or suppresses NET execution (19), as inhibition of mTOR signaling (autophagy inducer) enhanced NETs (49, 50), while others reported the opposite (51). In our study, wortmannin- or Baf A1–mediated autophagy inhibition exacerbated S1P-induced NETosis (Figure 8 and Supplemental Figure 8). These conflicting data may easily be reconciled. First, NET inducers (PMA, fMLP, LPS) and their concentrations differed in each study (49–51). Second, despite some common mechanisms of NET formation (22), multiple pathways can independently trigger NETosis. As reported (52, 53), caspase-11–deficient mice were resistant to NETs, indicating caspase-11 activation was indispensable for NETs in the LPS model. While S1PR2 signaling was implicated in caspase-11–dependent macrophage pyroptosis in one sepsis model (54), others found *CC1* regulated LPS-driven NLRP inflammasome and caspase-1 activation (21). Hence, we first thought *CC1* might control NETosis in a caspase-1/11–dependent manner. However, S1P-induced NET formation in *CC1*-deficient neutrophils was inflammasome independent (Supplemental Figure 14). On the contrary, lysosomal proteases, such as CathB and CathD, were diminished (Figure 8), while caspase-11 cleavage was blocked in Baf A1–treated neutrophils (Supplemental Figure 15). This indicates that autophagy inhibition suppressed NETosis driven by caspase-11/inflammasome activation while accelerating NETosis in which autophagy induction/lysosomal quality was essential for preventing NETs.

Although CathB inhibition has been linked with the suppression of LPS-driven proinflammatory response in *CC1*-KO neutrophils (21), we found CathB blockade increased H3Cit levels in WT and *CC1*-KO neutrophil cultures (Figure 8). In addition, S1PR2 ligation partially restored CathD and decreased H3Cit levels under Baf A1 preconditioning. These results imply S1PR2 ligation antagonized the V-ATPase function and attenuated NET formation. Intriguingly, though pretreatment with wortmannin (an early stage autophagy inhibitor) exacerbated S1P-induced NETs (Supplemental Figure 8), adjunctive S1PR2 ligation attenuated H3Cit expression to a lower level than that after S1P stimulation alone. Several reports have documented that PI3K, which is one of the wortmannin targets, regulates crosstalk between V-ATPase and F-actin in osteoclasts (55) or that PI3K has been involved in “regulated V-ATPase assembly” in dendritic cells (56). It has been shown in some studies that the S1PR2/S1PR3 axis regulated Na^+^/K^+^ ATPase in the hepatocellular carcinoma cell line (57, 58), while others have indicated that Na^+^/K^+^ ATPase might serve as an alternative for V-ATPase in astrocytes (59). Considering V-ATPase inhibitor (Baf A1) enhanced NET formation in parallel with depletion of lysosomal proteins (Figure 8) while S1PR2 ligation restored these proteins, we assumed S1PR2/3 signaling is involved in the reciprocal regulation of V-ATPase. Indeed, others documented the role of V-ATPase in LC3 lipidation and autophagy (60, 61). More studies are needed to determine whether and how the neutrophil S1PR2/S1PR3 axis directly or indirectly affects ATPase to determine the lysosomal function. With lysosomal quality being essential for NETosis and autophagy inhibition leading to lysosomal dysfunction (62), it was not surprising that activated ATG5-deficient neutrophils undergo NETosis (63). Future studies should address the impact of S1P stimulation upon NETs in autophagy-related protein-deficient (e.g., ATG5, ATG7, or LC3B) mice.

To directly assess the role of neutrophil CC1 signaling in IRI-OLT, we used a new PMN*^DTR^* transgenic murine model allowing selective and inducible ablation of native neutrophils (20). Indeed, reconstitution of PMN*^DTR^* mice with adoptively transferred *CC1*-deficient (*CC1*-KO), but not *CC1*-proficient (WT), neutrophils exacerbated liver IRI, which was accompanied by enhanced NET formation (Figure 8). These results highlight the pathogenic function of *CC1*-null neutrophils and document the ability of neutrophil *CC1* to recreate homeostasis in IR-stressed liver. The experimental sequence of our adoptive cell transfer and liver screening was essential (Supplemental Figure 10), as neutrophil depletion in PMN^DTR^ mice was transient and blood neutrophils started reappearing 2 days after DT injection.

S1PR2 signaling has been demonstrated to drive NASH (12, 64), while genetic S1PR3 ablation predisposes HFD-fed mice to inflammation/steatosis (65). This might be one of the reasons that the S1PR2/S1PR3 imbalance prompted *CC1*-deficient mice to readily develop steatohepatitis (14). Indeed, in our NASH-mimicking model, HFD-fed mice were more sensitive to IR-triggered OLT damage, concomitant with lower neutrophil *CC1* expression and enhanced NETosis, as compared with regular diet–fed WT mice (Hirao, unpublished observations). With 8 cases in our clinical cohort, we could not conclude that the “low-CC1/high-S1PR2” OLT phenotype was prevalent in patients. Even though S1P is one of many potential neutrophil activation stimuli in IRI-OLT, in NASH, expected to become the next global epidemic, liver grafts are exposed to high S1P levels, while those with S1P-enriched steatotic livers remain at very high risk for developing NETosis (66).

*CC1*-null mice exhibited enhanced NET formation in the IR-stressed OLT and in distal organs, such as lungs. As neutrophils in the lungs of WT and *CC1*-KO mouse OLT recipients expressed increased ICAM-1 levels (Hirao, unpublished observations), we assume that, in addition to enhanced susceptibility to NET formation in response to circulating S1P, ICAM-1–overexpressing *CC1*-deficient neutrophils might be more “toxic” in NET-driven lung injury. Whether these neutrophils reversely transmigrate from the liver (one of the newly discovered neutrophil functions) (67) awaits future study. In agreement with a previous report (68), neutrophils and platelets colocalized in IR-stressed liver and distant lungs (Hirao, unpublished observations), suggesting that *CC1*-deficient neutrophils may interact with platelets to cause tissue damage at the periphery, such as NET-driven lung thrombosis.

In conclusion, we have identified what we believe to be a novel regulatory mechanism by which the neutrophil CC1-L isoform controls the S1PR2/S1RP3 axis and NETosis via an autophagy pathway. As a checkpoint regulator of IR stress and sterile inflammation, neutrophil CC1-L may serve as a biomarker of NET formation, hence guiding early postoperative management and decision making for therapeutic intervention in OLT recipients.

## Methods

### Clinical liver transplant study.

We performed a retrospective analysis of 55 adult patients (≥18 years) who underwent OLT (May 2013–August 2015). All the recipients received standard of care and immunosuppressive therapy, as specified by UCLA protocols. Recipients who underwent retransplantation were excluded. Donor livers, procured from donation after brain or cardiac death with standardized techniques, were perfused with and stored in UW solution (Niaspan, Bristol Myers Squibb). Protocol Tru-Cut needle Bx, were obtained from the left liver lobe at about 2 hours after portal reperfusion (prior to the abdominal closure). Cold ischemia time was defined as the time from perfusion of the donor liver with UW solution to its removal from cold storage. Warm ischemia time was defined as the time from cold-storage removal to the establishment of graft reperfusion. Recipient blood was collected before and after OLT, and sALT/sAST levels were used to evaluate liver function. The plasma samples collected at 2 hours after reperfusion were available from 39 cases (August 2014–August 2015). EAD was defined by the presence of one or more of the following: bilirubin level of 10 mg/dl or greater on POD7, PT/INR of 1.6 or greater on POD7, or aspartate transferase (AST) or alanine transaminase (ALT) level greater than 2,000 U/L within the first 7 days.

### Animals.

WT mice (Jackson Laboratory) and mice with global *CC1* ablation (*CC1*-KO; courtesy of M. Kujawski and J. Shively, Beckman Research Institute, City of Hope, Duarte, California, USA; originally generated by N. Beauchemin, McGill University, Montreal, Canada) on a C57BL/6 background and 6 to 8 weeks of age were used. Mrp8cre^Tg^ (B6.*CG*-Tg [S100A8-cre,-EGFP]1Ilw/J; stock no. 021614) mice and ROSA26iDTR (C57BL/6-*Gt[ROSA]26Sortm1[HBEGF]Awai/J*; stock no. 007900) mice were purchased from The Jackson Laboratory and crossed to generate DT-inducible PMN-depleted mice (MRP8-Cre^+^; ROSA-iDTR), as reported (20). Animals were housed in the UCLA animal facility under pathogen-free conditions.

### Mouse models of liver IRI.

We used a mouse model of ex vivo hepatic cold storage followed by liver transplantation (4). To focus on recipient-derived CC1 neutrophil function while avoiding host alloimmune responses, donor livers (WT) stored in UW solution (4°C/18 hours) were transplanted to syngeneic mouse recipients. Liver graft and serum samples were collected at 6 hours after reperfusion, the peak of hepatocellular damage in this model. The sham-treated group underwent the same procedures except for OLT.

We used a DT-inducible neutrophil depletion (PMN*^DTR^*) transgenic mouse model (20). PMN*^DTR^* mice were pretreated with DT (500 ng/mouse, i.p., D0564, MilliporeSigma), and 24 hours after native PMN depletion (assessed by FACS), neutrophils isolated from WT or *CC1*-KO donor mice were adoptively transferred into PMN*^DTR^* mice (3 × 10^7^ cells/mouse, i.v.), followed by warm hepatic ischemia insult (90 minutes) as described (69). Some PMN*^DTR^* mice were pretreated with CI-amidine (50 mg/kg, s.c.; 10599, Cayman Chemical) at 1 hour prior to the ischemia insult. Blood and liver tissue samples were collected at 6 hours after reperfusion.

### Hepatocellular function assay.

sAST/sALT levels were measured with Infinity AST/ALT Liquid Stable Reagent (Thermo Scientific) and validated with Validate GC3 (Maine Standards Company).

### Liver histology/IRI grading.

Formalin-fixed, paraffin-embedded liver sections (5 μm) were stained with H&E, and the severity of hepatic IR damage was graded using Suzuki’s criteria (70).

### TUNEL assay.

Cell death in liver sections (5 μm) was screened using the In Situ Apoptosis Detection Kit (MK500, Clontech) according to the manufacturer’s protocol. Results were scored semiquantitatively by blinded counting of the number of positive cells in 10 HPF/section.

### ELISA assay.

Serum concentration of S1P in mice (MBS2700637, MybioSource) and plasma concentration of NE (DY9167-05, R&D Systems Inc.) and MPO (440007, BioLegend) in humans were measured by ELISA according to the manufacturers’ protocols.

### Neutrophil isolation/culture.

Bone marrow–derived neutrophils were isolated from femurs/tibias by using the EasySep Mouse Neutrophil Enrichment Kit (19762, Stem Cell Technologies). Freshly isolated neutrophils were cultured (1.5 × 10^6^ cells/mL in RPMI supplemented with 2% FBS and Antimycotic; 15240062, Thermo Fisher) and stimulated with S1P (SML2709, Millipore Sigma) or LPS (L5293, Millipore Sigma). In some experiments, neutrophils were pretreated with S1PR2 antagonist (JTE-013, 10009458, Cayman Chemical), S1PR3 antagonist (TY 52156, 19119, Cayman Chemical), autophagy antagonists, wortmannin (12-325, Fisher Scientific) or Baf A1 (54645S, Cell Signaling Technology), or CathB inhibitor (CA-074, 4846, Bio-Techne Corp.).

### WB assay.

Proteins were extracted from tissue/cell samples, and their concentration was measured using the BCA Protein Assay Kit (Thermo Scientific). Then, proteins were electrophoresed in denaturing conditions, blotted, incubated with primary Abs and secondary HRP-conjugated Abs, and developed. Primary Abs used in this study are listed in Supplemental Table 4. To compare target protein expression in multiple human OLT samples, densitometry quantification was conducted, as reported (4).

### Quantitative RT-PCR analysis.

RNA was extracted with the NucleoSpin RNA Kit (740955, Takara Bio), and reverse transcription was performed for cDNA synthesis with the PrimeScript RT Reagent Kit (RR037, Takara Bio). Quantitative PCR was performed using QuantStudio 3 (Applied Biosystems). Primer sequences are listed in Supplemental Table 5. The expression of the target gene was normalized to the housekeeping 18S, HPRT, or GAPDH.

### Hepatocyte isolation/culture.

Primary mouse hepatocytes were isolated by a 2-stage collagenase perfusion method as described (71). The hypoxia condition was induced by an oxygen adjustable incubator (Heracell 150i,51026280, Thermo Fisher Scientific). Hypoxia and reoxygenation were conducted as previously reported (72).

### Flow cytometry.

Mouse PBL samples or cultured neutrophils were first incubated with anti-mouse CD16/32 Abs (clone: 93 BioLegend) to block Fc-mediated nonspecific Ab binding. Cells were then stained with the fluorochrome-conjugated Abs, as listed in Supplemental Table 4. Multiparameter flow cytometric analysis was performed using an LSR Fortessa X-20 SORP (BD Bioscience), and results were analyzed using BD FACSDiva software at the UCLA Jonsson Comprehensive Cancer Center (JCCC) and the Center for AIDS Research Flow Cytometry Core Facility (UCLA).

### IF/immunohistochemistry.

Frozen mouse liver samples (5 μm) were stained with rat anti-CD68 Abs, rat anti-Ly6G Abs, rabbit anti-H3Cit Abs, sheep anti-CC1 Abs, or rabbit anti-S1PR2 Abs. Hepatic Ly6G^+^ cells were scored semiquantitatively by blinded counting of cells in 10 HPF/sections (×400). Isolated neutrophils were stained using sheep anti-CC1 Abs, rat anti-Ly6G Abs, goat anti-MPO Abs, or rabbit anti-H3Cit Abs. Signals were visualized with secondary Alexa Fluor Abs. For S1P detection, formalin-fixed, paraffin-embedded liver tissues (5 μm thickness) were stained using mouse anti-S1P Abs with the M.O.M. (Mouse on Mouse) ImmPRESS HRP (peroxidase) Polymer Kit (MP-2400, Vector Laboratories).

### NET visualization/quantification.

Eight-well Chamber Slides (1256522, Fisher) were coated with poly-l-lysine solution (A005C, MilliporeSigma), and neutrophils were seeded (2 × 10^5^ cells/well). After the induction of NETs, each well was washed with PBS 3 times and fixed with 2% paraformaldehyde at 4°C overnight. Fixed cells were stained with anti-CC1, anti-Ly6G, anti-MPO, or anti–histone H3. DNA was counterstained with Hoechst 33342 (H3570, Invitrogen). NET-positive neutrophils were blindly counted (×200).

For additional information, see Supplemental Methods.

### Statistics.

For mouse experiments, comparisons between 2 groups were assessed using Student’s *t* test or 1 way-ANOVA followed by Tukey’s honestly significant difference (HSD) test. For human data, continuous values were analyzed by Mann-Whitney *U* test and categorical variables by Fisher’s exact test. All *P* values were determined by 2-tailed tests, and *P* < 0.05 was considered statistically significant.

### Study approval.

All human studies were approved by the UCLA Institutional Research Board (IRB protocol 13-000143) and written, informed consent was received from participants prior to inclusion in the study. All mouse experiments were approved by the UCLA Animal Research Committee (ARC 1999-094). Animals received humane care according to the criteria outlined in the *Guide for the Care and Use of Laboratory Animals* (NIH publication 86-23, revised 1985).

## Author contributions

HH, YZ, and JWKW conceived and designed the study. HH, HK, KJD, KN, and KK acquired experimental data. HK performed mouse surgical procedures. HH, KK, and FMK analyzed clinical data. HH, HK, KJD, KN, KK, YZ, DGF, FMK, and JWKW discussed the manuscript. HH and JWKW drafted the manuscript. JWKW obtained funding. All authors read and edited the manuscript.

## Supplementary Material

Supplemental data

## Version 1 02/01/2023

Electronic publication
