# Supplementary material for: Neutrophil CEACAM1 determines susceptibility to NETosis by regulating the S1PR2/S1PR3 axis in liver transplantation
Source: J Clin Invest. 2023 Feb 1;133(3):e162940. doi: 10.1172/JCI162940 (PMC9888387; doi:10.1172/JCI162940)
Supplement: Supplemental data [file jci-133-162940-s140.pdf]

Neutrophil CEACAM1 Determines the Susceptibility to NETosis by Regulating the S1PR2/S1PR3 Axis in Liver Transplantation

Hirofumi Hirao[1*], Hidenobu Kojima[1*], Kenneth J. Dery[1], Kojiro Nakamura[1,3], Kentaro Kadono[1], Yuan Zhai[1], Douglas G. Farmer[1], Fady M. Kaldas[1] and Jerzy W. Kupiec-Weglinski[1,2]

[1] Dumont-UCLA Transplantation Center, Department of Surgery, Division of Liver and Pancreas Transplantation; [2] Department of Pathology and Laboratory Medicine, David Geffen School of Medicine at UCLA, Los Angeles, CA 90095; [3] Department of Surgery, Hepato-Biliary-Pancreatic Surgery and Transplantation, Kyoto, Japan

Authorship note: *These authors contributed equally

Table of Contents

Supplemental Table 1……………………………………………………………………………..3

Supplemental Table 2……………………………………………………………………………..4

Supplemental Table 3……………………………………………………………………………..5

Supplemental Table 4……………………………………………………………………………..6

Supplemental Table 5……………………………………………………………………………..7

Supplemental Figure 1……………………………………………………………………………8

Supplemental Figure 2……………………………………………………………………………9

Supplemental Figure 3……………………………………………………………………………10

Supplemental Figure 4……………………………………………………………………………11

Supplemental Figure 5……………………………………………………………………………12

Supplemental Figure 6……………………………………………………………………………13

Supplemental Figure 7……………………………………………………………………………14

Supplemental Figure 8……………………………………………………………………………15

Supplemental Figure 9……………………………………………………………………………16

Supplemental Figure 10…………………………………………………………………………..17

Supplemental Figure 11…………………………………………………………………………..19

Supplemental Figure 12…………………………………………………………………………..20

Supplemental Figure 13…………………………………………………………………………..21

Supplemental Figure 14…………………………………………………………………………..22

Supplemental Figure 15…………………………………………………………………………..23

Supplemental Methods/Results/Reference....………………..……………………………….24

Experimantal data of murine OLT			
	**WT→WT**	**WT→*CC1-KO***	**p value**
sAST (IU/L)	4100±484	7029±1105	0.0430
sALT (IU/L)	5827±696	9356±1099	0.0246
Suzuki's histological score	5.8±0.3	7.7±0.7	0.0310
$TUNEL^+$ cells (/field)	38.0±6.0	97.9±15.8	0.0077
$Ly6G^+$ cells (/field)	45.4±4.7	63.5±5.2	0.0415

Table S1

Recipients' and surgical parameters			
**Variables**	**low CAECAM1-L/Cathepsin G n=28**	**high CEACAM1-L/Cathepsin G n=27**	**p value**
Age (years)	57 (23-73)	52 (29-68)	0.066
Gender (M/F)	20 (71.4%) / 8 (28.6%)	17 (62.9%) / 10 (37.1%)	0.573
Race			0.865
White	18 (64.2%)	12 (44.4%)	
Hispanic	6 (21.4%)	14 (51.9%)	
Black	0	0	
Asian	2 (7.2%)	0	
Others	2 (7.2%)	1 (3.7%)	
BMI (kg/m^2)	26.9 (14.5-38.2)	30.0 (17.1-47.5)	0.304
Disease ehiology			0.417
Viral hepatitis	14 (50.0%)	12 (44.4%)	
EtOH	5 (17.8%)	4 (14.8%)	
Cryptogenic cirrhosis / NASH	4 (14.3%)	4 (14.8%)	
ALF	1 (3.6%)	0 (0%)	
Others	4 (14.3%)	7 (26.0%)	
ABO			
identical	28 (100%)	27 (100%)	N / A
MELD score	27.6 (9-42)	33.8 (16-44)	0.006
Pre-transplant AST (IU/L)	124.7 (23-1231)	91.2 (22-283)	0.308
Pre-transplant ALT (IU/L)	79.1 (11-617)	46.6 (9-158)	0.833
T-Bil (g/dl)	11.6 (0.3-59.1)	16.4 (0.3-49.2)	0.099
PT-INR	1.70 (1.0-3.0)	2.02 (1.0-3.2)	0.013
Pre-operative hospital stay (days)	12.6 (0-78)	14.0 (0-55)	0.323
CIT (min)	464.7 (120-1213)	494.7 (285-818)	0.202
WIT (min)	53 (29-75)	58 (38-80)	0.325

Table S2

Donors' parameters

**Variables**	**low CAECAM1-L/Cathepsin G n=28**	**high CEACAM1-L/Cathepsin G n=27**	**p value**
Age (years)	45 (16-66)	34 (13-67)	0.015
Gender (M/F)	13 (46.4%) / 15 (53.6%)	15 (55.5%) / 12 (44.5%)	0.593
Race			0.793
White	16 (57.1%)	16 (59.2%)	
Hispanic	10 (35.7%)	8 (29.6%)	
Black	1 (3.6%)	1 (3.7%)	
Asian	1 (3.6%)	2 (7.5%)	
Others	0	0	
BMI (kg/m^2)	27.6 (19.7-42.6)	25.9 (13.4-38.4)	0.2629
Pre-transplant AST (IU/L)	91.9 (11-749)	73.4 (10-347)	0.256
Pre-transplant ALT (IU/L)	82.0 (8-887)	95.1 (11-1184)	0.249
T-Bil (g/dl)	0.7 (0.3-2.1)	1.1 (0.2-4.9)	0.183
PT-INR	1.31 (1.0-2.0)	1.32 (1.0-2.2)	0.889
DCD	3 (10.7%)	0	0.236

Table S3

Primary Antibodies Used For Western Blot and Immunostaining

Ab name	catalog	host	clone	application	company
PAD4	684202	mouse	O94H5	WB	Biolegend
MPO	AF3667	goat	n/a	WB/ICC	R&D
Histone H3 (citrulline R2 + R8 + R17)	ab5103	rabbit	n/a	WB/IHC (tissue)/ICC	abcam
Histone H3 (citrulline R2 + R8 + R17)	ab281584	rabbit	RM1001	WB (vitro/serum)	abcam
S1PR2	ab235919	rabbit	n/a	WB/IHC/ICC	abcam
EDG3/S1P3	ab108370	rabbit	n/a	WB	abcam
CEACAM1	AF6480	sheep	n/a	IHC/ICC	R&D
CEACAM1	MAB6480	rat	723629	WB/ICC	R&D
NLRP3	MAB7578	rat	768319	WB	R&D
Caspase-1	AG-20B-0042-C100	mouse	Casper-1	WB	AdipoGen
Caspase-11	564971	rat	17D9	WB	BD
Gasdermin D	ab209845	rabbit	EPR19828	WB	abcam
Vinculin	13901S	rabbit	E1E9V	WB	Cell signaling
Ly6G	551459	rat	1A8	IHC/ICC	BD
CD68	MCA1957	rat	FA-11	IHC	Bio-Rad
Cathepsin D	AF1029	goat	n/a	WB/ICC	R&D
S1P	Z-P300	mouse	LT1002	IHC	Echelon
SPHK1	658302	mouse	1A5SC	WB	Biolegend
β-actin	sc-47778	mouse	C4	WB	Santa Cruz
LC3B	ab192890	rabbit	EPR18709	WB	abcam
SQSTM1 / p62	ab109012	rabbit	EPR4844	WB/ICC	abcam
PI3 Kinase Class III (Vps34)	4263S	rabbit	D9A5	WB	Cell signaling
Beclin-1	3495S	rabbit	D40C5	WB	Cell signaling
Ly-6G	87048	rabbit	E6Z1T	WB	Cell signaling
Cathepsin B	31718S	rabbit	D1C7Y	WB	Cell signaling
HMGB1	ab79823	rabbit	EPR3507	WB	abcam
CEACAM-1 long isoform specific antibody	n/a	rabbit	n/a	WB	gift from Dr. Shively, City of Hope
APC anti-mouse/human CD11b	101212	rat	M1/70	FCM	Biolegend
PE anti-mouse Ly-6G	127608	rat	1A8	FCM	Biolegend
Alexa Fluor® 488 anti-mouse CD66a (CEACAM1a)	134526	mouse	MAb-CC1	FCM	Biolegend

WB: western blot, ICC: immunocytochemistry, IHC: immunohistochemistry, FCM: flow cytmetry

Table S4

Primer Sequences Used for Real-Time Quantitative PCR

species	Gene	Forward	Reverse
Human	*TLR4*	5'-AGACCTGTCCCTGAACCCTAT-3'	5'-CGATGGACTTCTAAACCAGCCA-3'
	CD80	5'-AAACTCGCATCTACTGGCAAA-3'	5'-GGTTCTTGTACTCGGGCCATA-3'
	CD86	5'-CTGCTCATCTATACACGGTTACC-3'	5'-GGAAACGTCGTACAGTTCTGTG-3'
	CXCL10	5'-GTGGCATTCAAGGAGTACCTC-3'	5'-TGATGGCCTTCGATTCTGGATT-3'
	CD68	5'-GGAAATGCCACGGTTCATCCA-3'	5'-TGGGGTTCAGTACAGAGATGC-3'
	Cathepsin G	5'-GAGTCAGACGGAATCGAAACG-3'	5'-CGGAGTGTATCTGTTCCCCTC-3'
	CD28	QuantiTect Primer Assay (QT00001267)	
	CD4	QuantiTect Primer Assay (QT02401812)	
	IL17	5'-TCCCACGAAATCCAGGATGC-3'	5'-GGATGTTCAGGTTGACCATCAC-3'
	GAPDH	5'-GGAGCGAGATCCCTCCAAAAT-3	5'-GGCTGTTGTCATACTTCTCATGG-3'
Mouse	*TNFA*	5'-GCCTCTTCTCATTCCTGCTTGT-3'	5'-GATGATCTGAGTGTGAGGGTCTG-3'
	IL1B	5'-TGTAATGAAAGACGGCACACC-3'	5'-TCTTCTTTGGGTATTGCTTGG-3'
	IL6	5'-TGGCTAAGGACCAAGACCATCCAA-3'	5'-AACGCACTAGGTTTGCCGAGTAGA-3'
	MCP1	5'-CATCCACGTGTTGGCTCA-3'	5'-GATCATCTTGCTGGTGAATGAGT-3'
	CXCL1	5'-ACCCAAACCGAAGTCATAG-3'	5'-TTGTATAGTGTTGTCAGAAGC-3'
	CXCL2	5'-ACTTCAAGAACATCCAGAG-3'	5'-CTTTCCAGGTCAGTTAGC-3'
	CXCL10	5'-GCTGCCGTCATTTTCTGC-3'	5'-TCTCACTGGCCCGTCATC-3'
	Sphk1	5'-ACAGTGGGCACCTTCTTTC-3'	5'-CTTCTGCACCAGTGTAGAGGC-3'
	Sphk2	5'-ACCACTTATGAGGAGAATCG-3'	5'-CACCACGTGGTCCATACAGC-3'
	HPRT	5'-TCAACGGGGGACATAAAAGT-3'	5'-TGCATTGTTTTACCAGTGTCAA-3'
	18S	5'-CGCTTCCTTACCTGGTTGAT-3'	5'-GAGCGACCAAAGGAACCATA-3'

Table S5

Figure S1

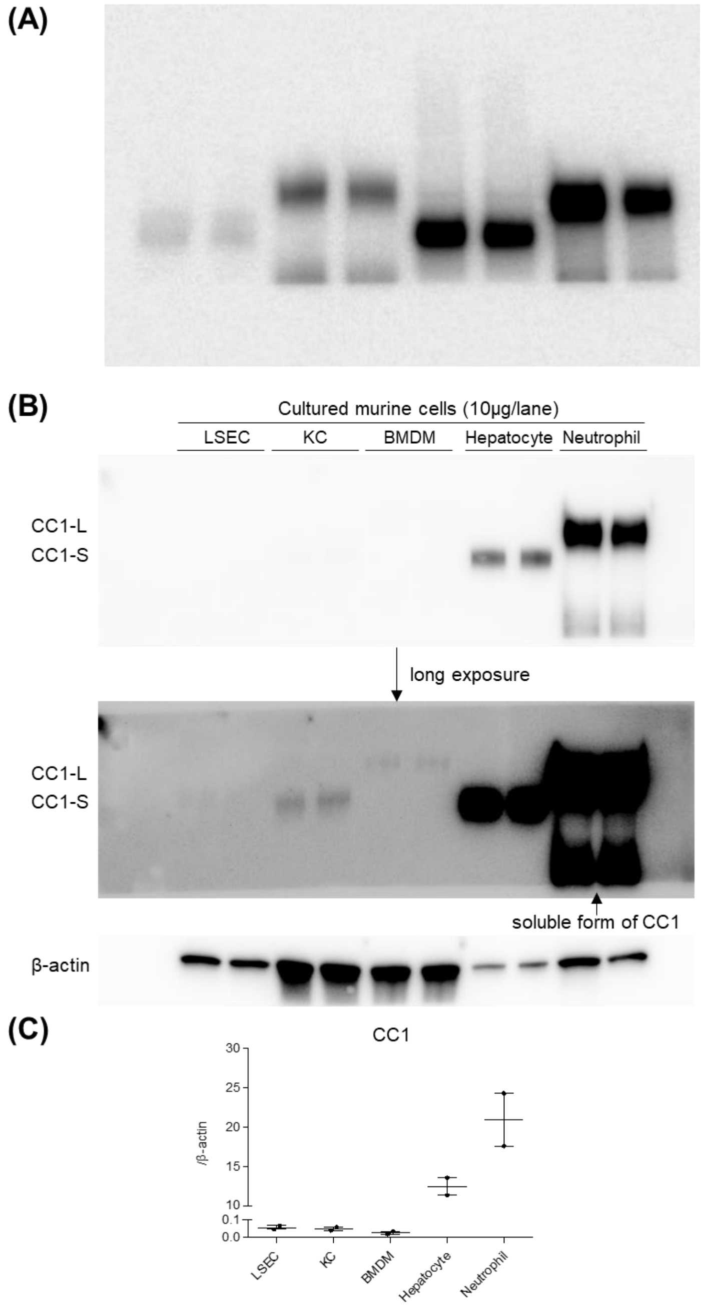

Suppl. Figure 1: Entire image of CC1 isoforms in cultured murine cells: (A) Full image of Figure 1B immunoblots. **(B)** Equal amount of protein (10μg) was applied in each lane and CC1 expression was analyzed by immunoblots. β-actin was used as a loading control. KC: Kupffer cell **(C)** The relative intensity of CC1 in each cell culture. Data shown as mean±SEM.

Figure S2

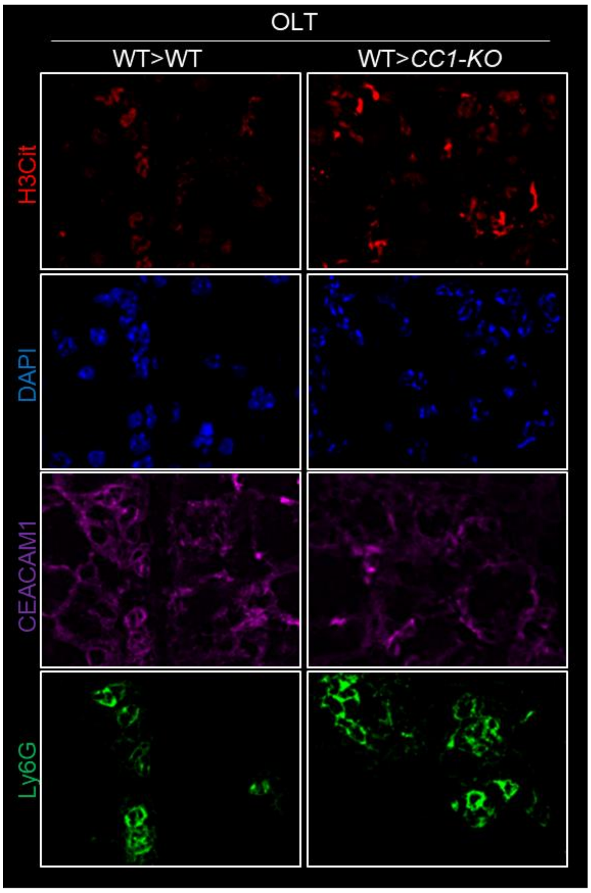

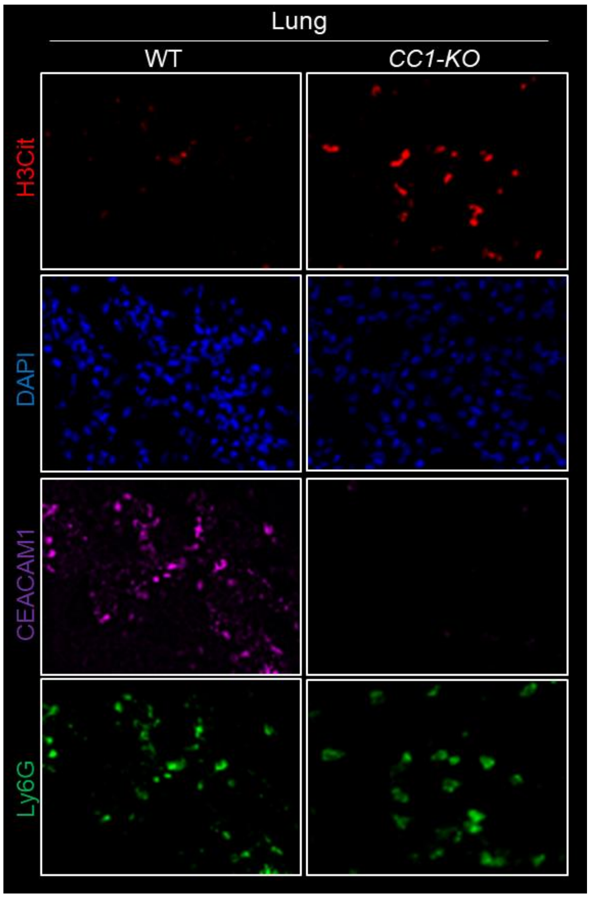

Suppl. Figure 2: Separate IF images of Figure 3

Figure S3

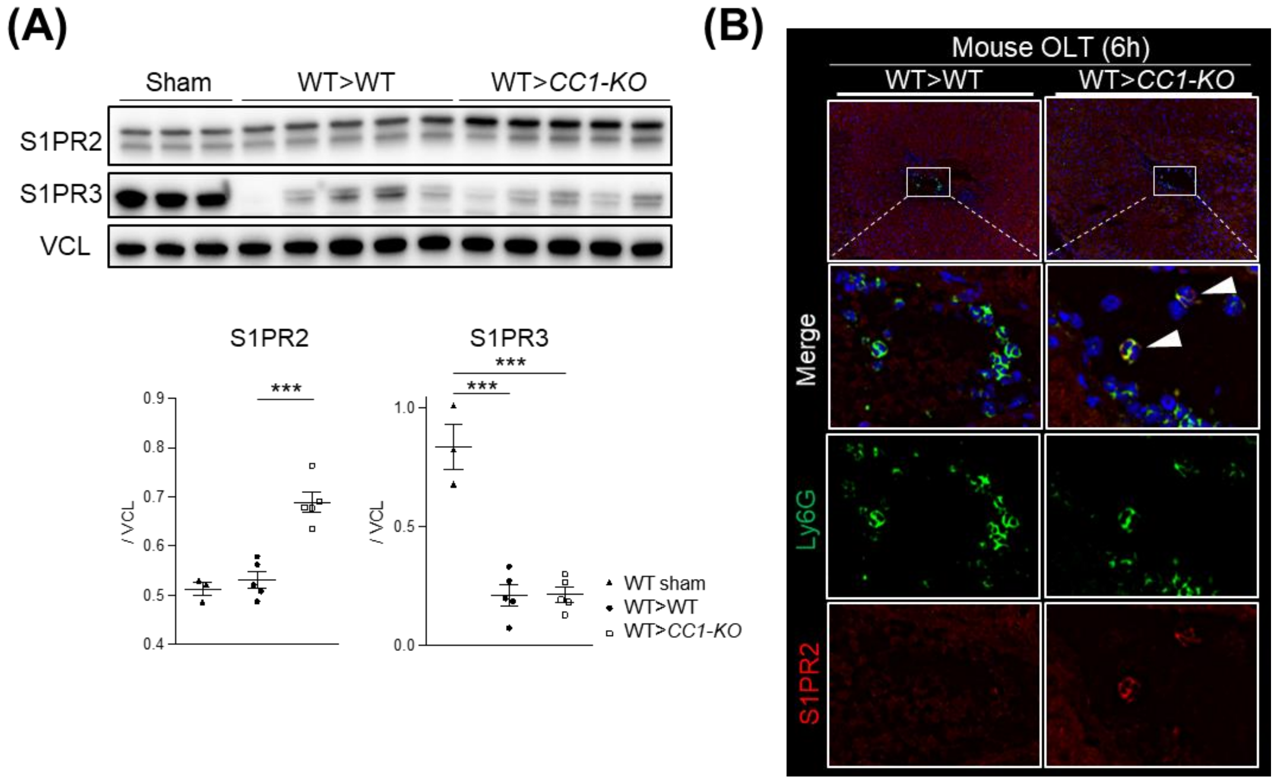

Suppl. Figure 3: Recipient *CC1* deficiency upregulates post-transplant hepatic *S1PR2* expression: (A) WB of S1PR2 and S1PR3 in sham and post-transplant livers. VCL was used as an internal control. Data shown as mean±SEM (***p<0.001, 1-way ANOVA followed by Tukey's HSD test, n=3-5/group). **(B)** Representative immunohistochemical staining of Ly6G (green) and S1PR2 (red) in OLT (original magnification x200).

Figure S4

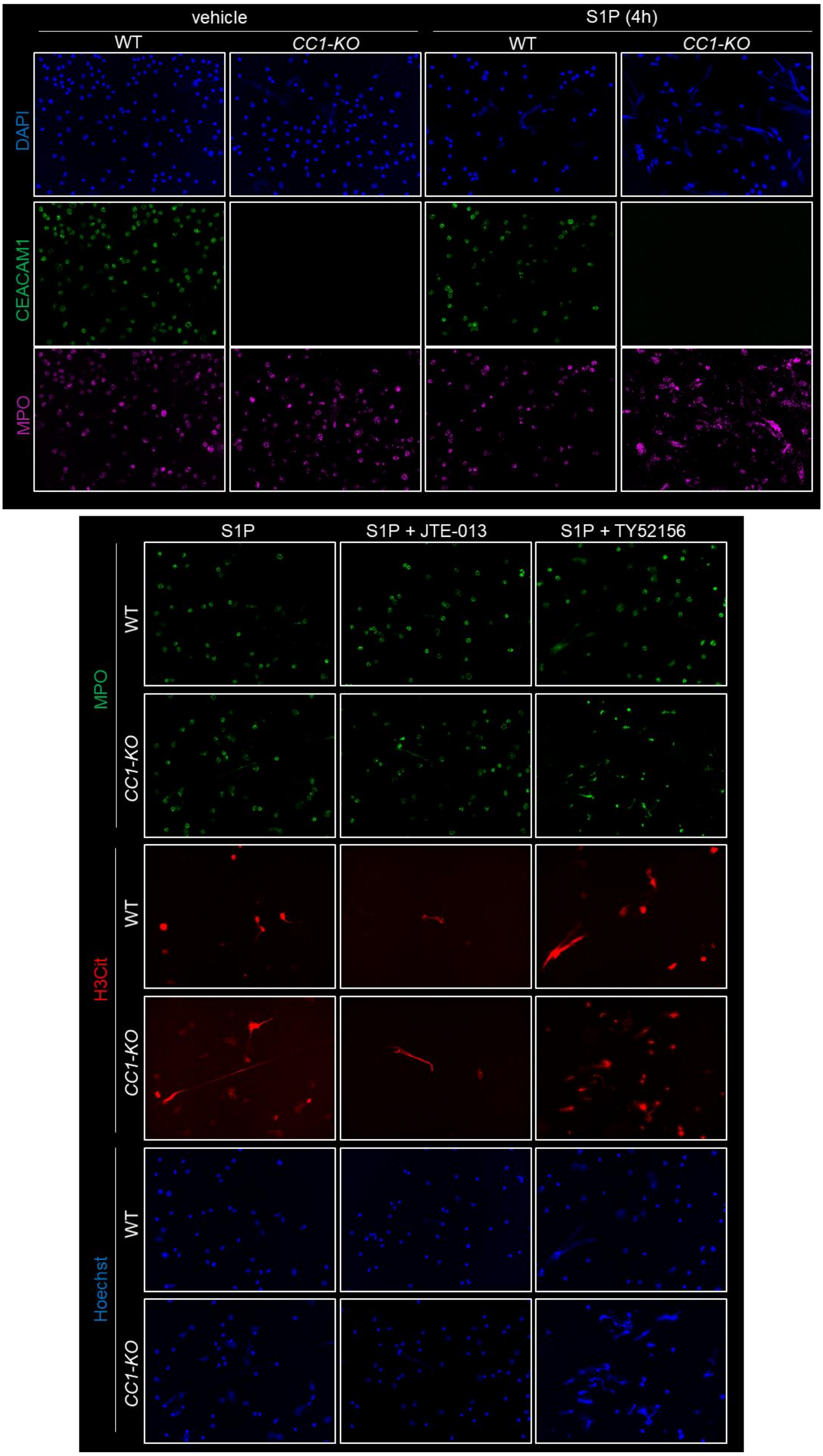

Suppl. Figure 4: Separate IF images of Figure 4

Figure S5

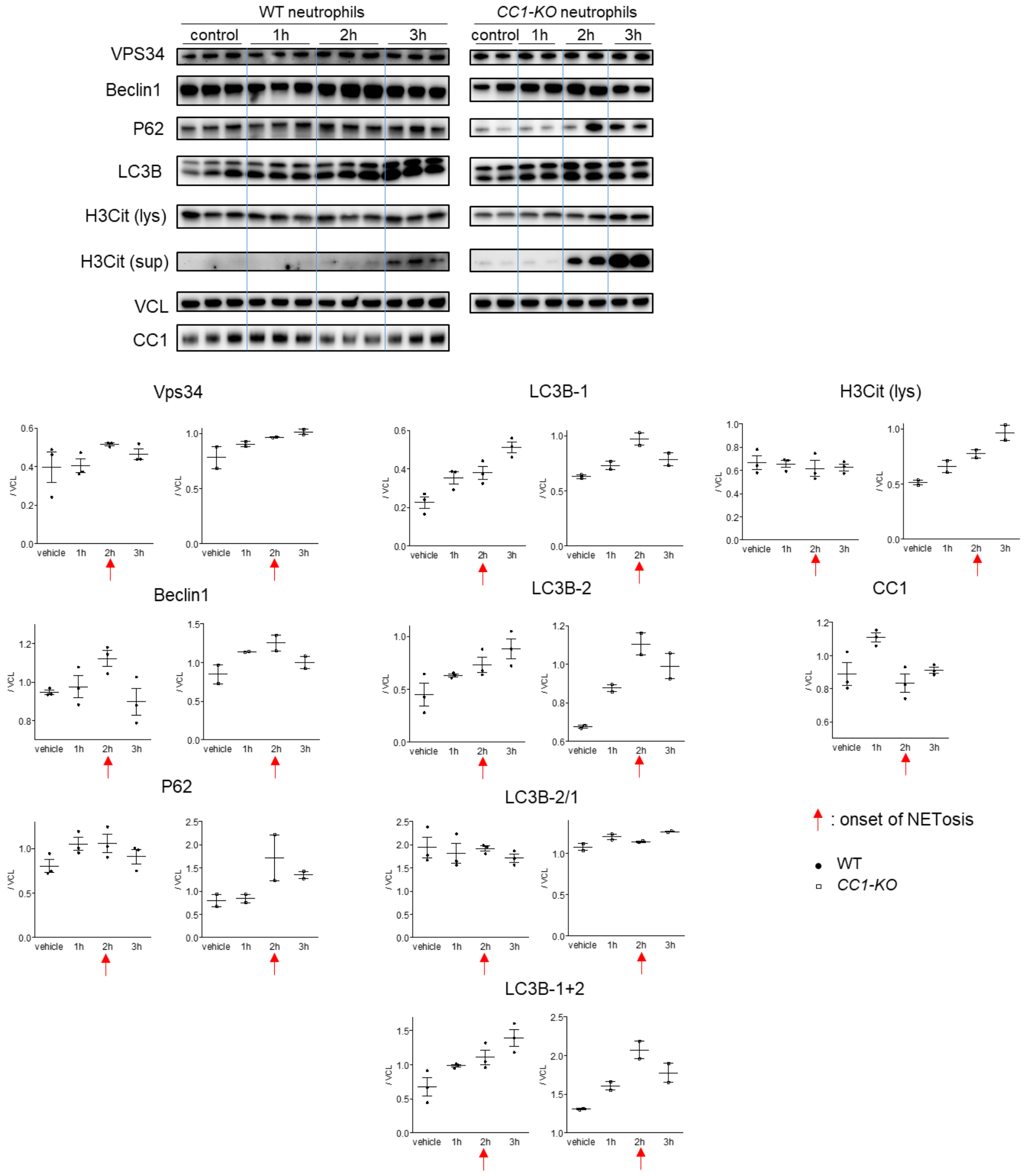

Suppl. Figure 5: Time-dependent alteration of autophagy-related proteins and Histone H3 citrullination (H3Cit) in response to S1P in WT and *CC1-KO* neutrophil cultures: WB of VPS34, Beclin1, p62, LC3B, H3Cit (lysate and supernatant) and *CC1* in WT or *CC1-KO* neutrophils stimulated with S1P (1μM; 0, 1, 2, 3h). VCL was used as an internal control. Data shown as mean±SEM (1-way ANOVA followed by Tukey's HSD test, *p<0.05, **p<0.01, ***p<0.0001, n=2-3/group). Red arrow indicates the onset of NETosis.

Figure S6

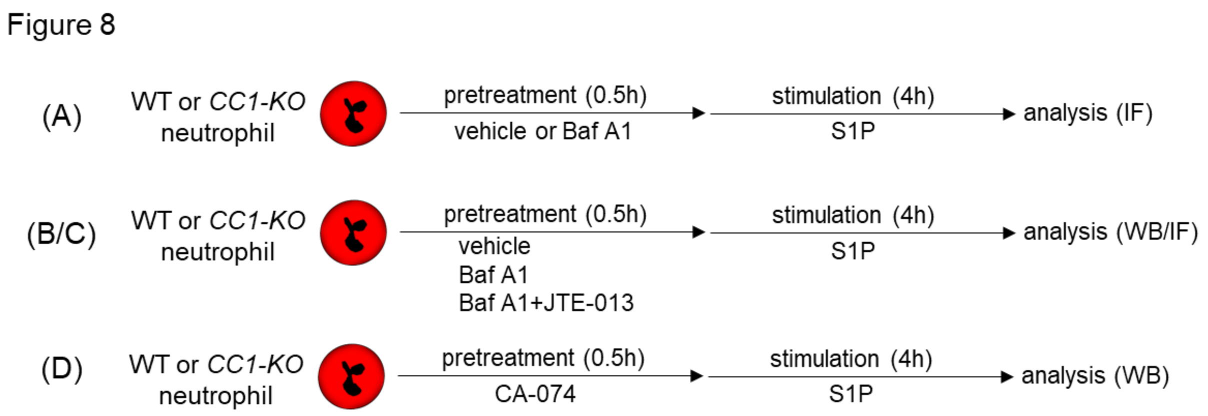

Suppl. Figure 6: Experimental scheme of vitro study in Figure 8

Figure S7

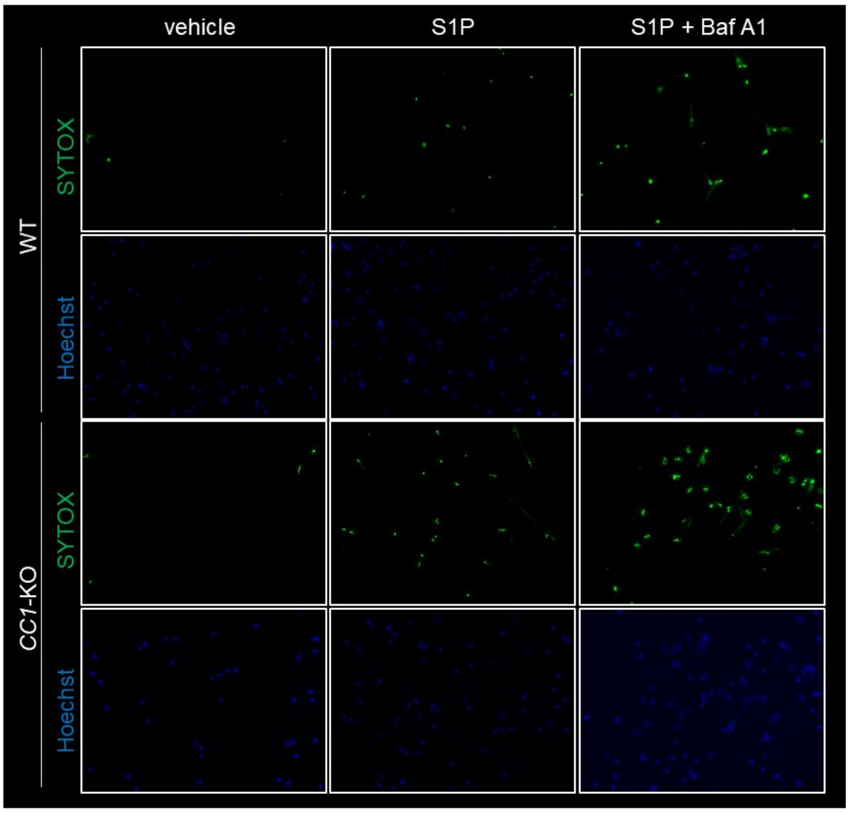

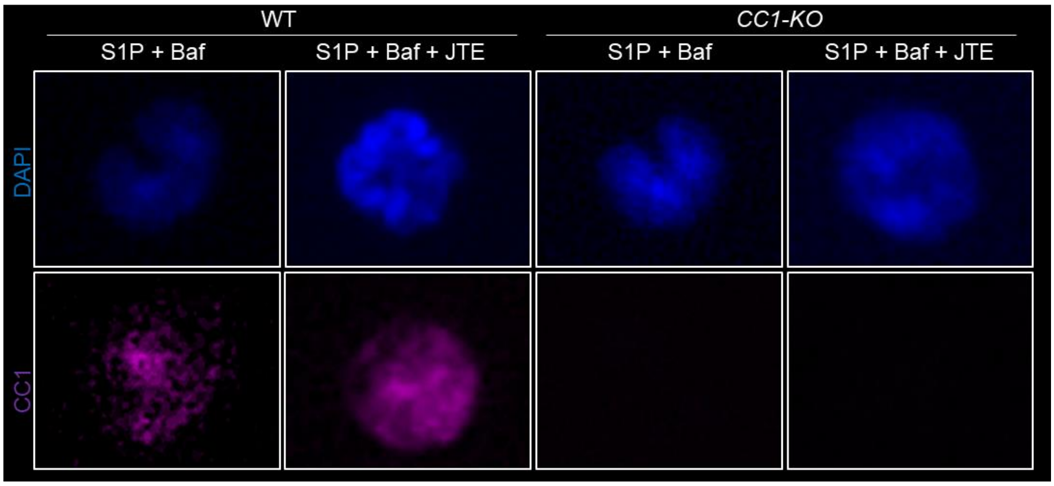

Suppl. Figure 7: Separate IF images of Figure 8

Figure S8

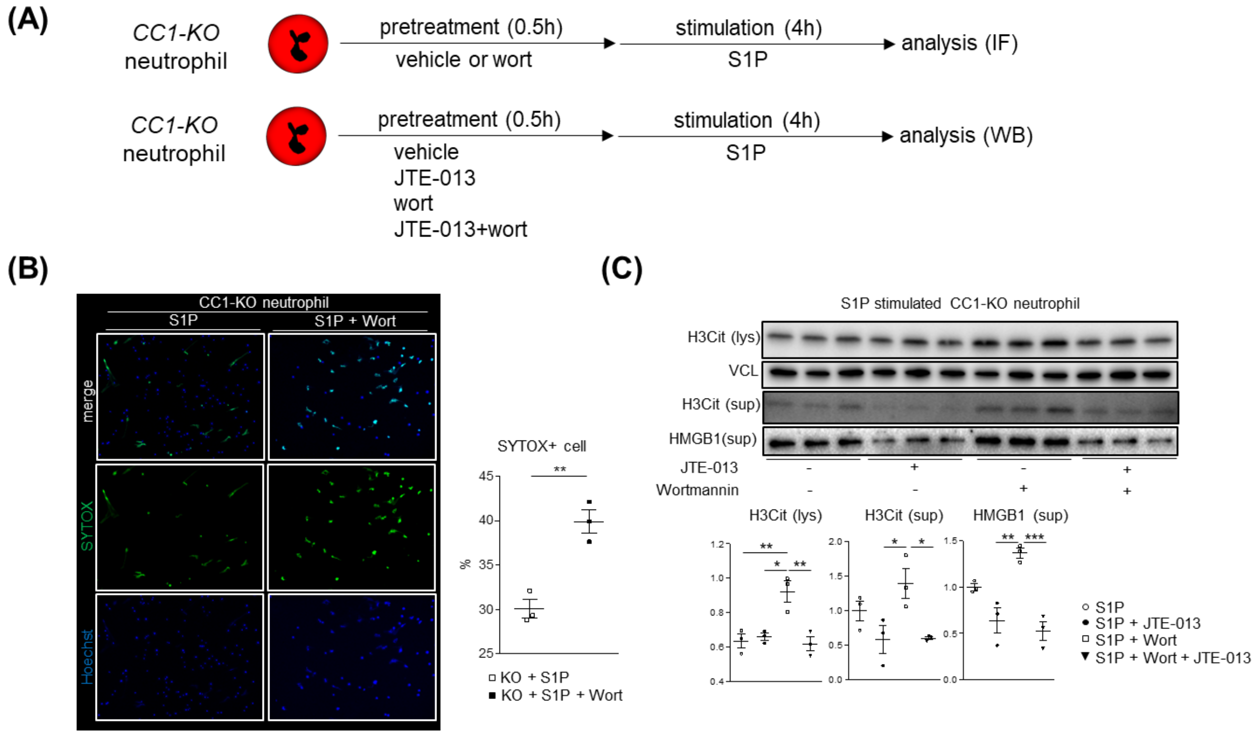

Suppl. Figure 8: S1PR2 ligation attenuated cit H3 levels and HMGB1 release in a wortmannin-conditioned environment: (A) Experimental scheme of vitro study. **(B)** Representative (n=3/group) IF images of SYTOX green (green) and Hoechst 33342 (blue) in *CC1-KO* neutrophils treated with S1P with or without wortmannin (100nM, 0.5h), and quantification of SYTOX green positive cells. Data are shown as mean±SEM (Student t-test, **p<0.01). Original magnification, x200. **(C)** WB of H3Cit (lysate and culture media) and HMGB1 (culture media) in *CC1-KO* neutrophils stimulated with S1P (1μM, 4h), vehicle or JTE-013 (10μM, 0.5h) or Wortmannin (100nM, 0.5h) or JTE-013 plus Wortmannin (10μM/100nM, 0.5h) pretreatment. VCL was used as an internal control. Data are shown as mean±SEM (1-way ANOVA followed by Tukey's HSD test, *p<0.05, **p<0.01, n=3/group).

Figure S9

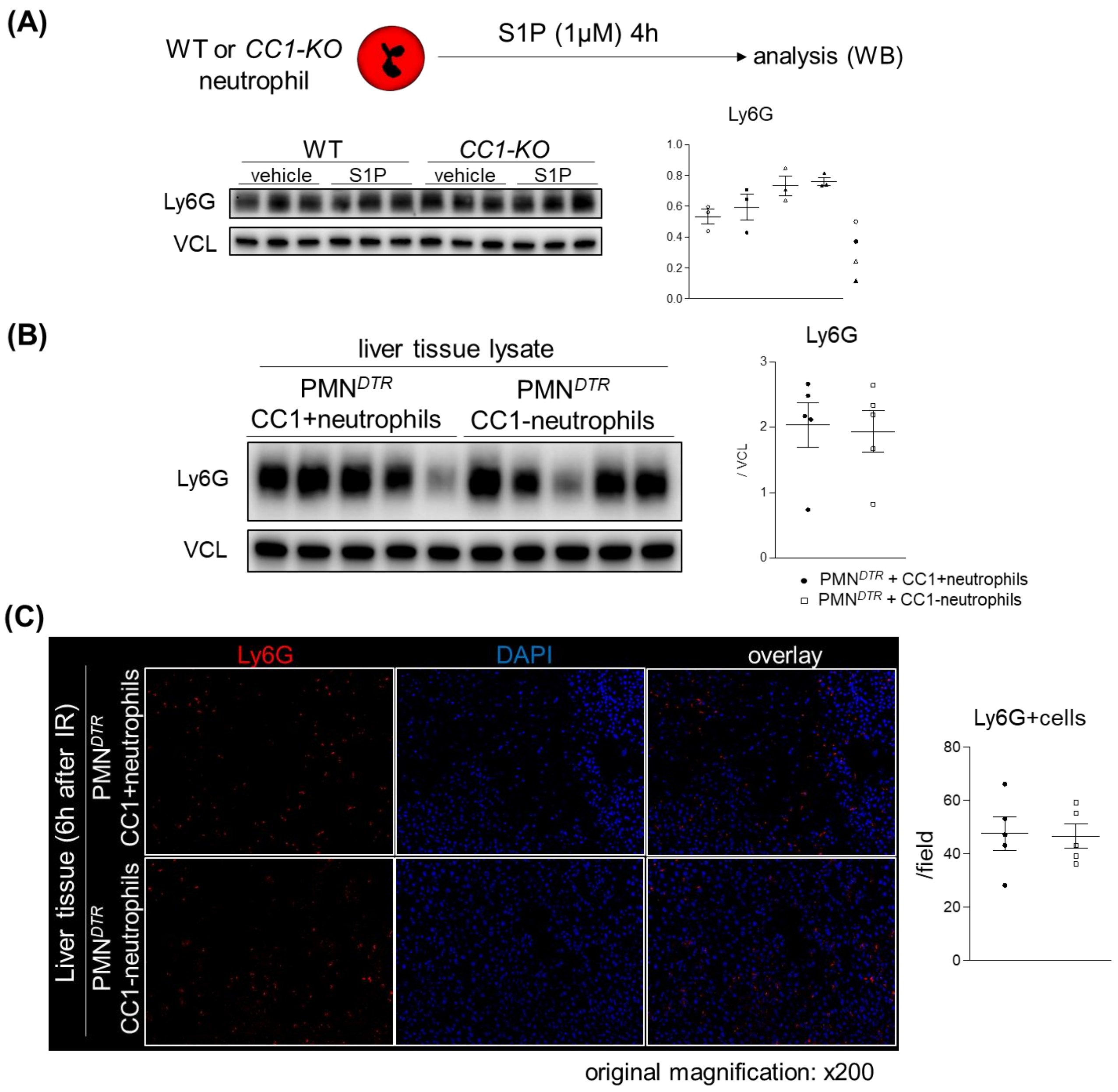

Suppl. Figure 9: The comparable number of neutrophils infiltrated post-IR livers in PMN^{DTR} mice: (A) WB of Ly6G expression in WT or *CC1-KO* neutrophils stimulated with S1P (1μM, 4h). VCL was used as an internal control. Data shown as mean±SEM. **(B)** WB of Ly6G expression in the livers of PMN^{DTR} mice 6h after reperfusion. VCL was used as an internal control. Data shown as mean±SEM. **(C)** Representative immunofluorescence of Ly6G in post-IR livers and frequency of infiltrating Ly6G+ cells. Data shown as mean±SEM.

Figure S10

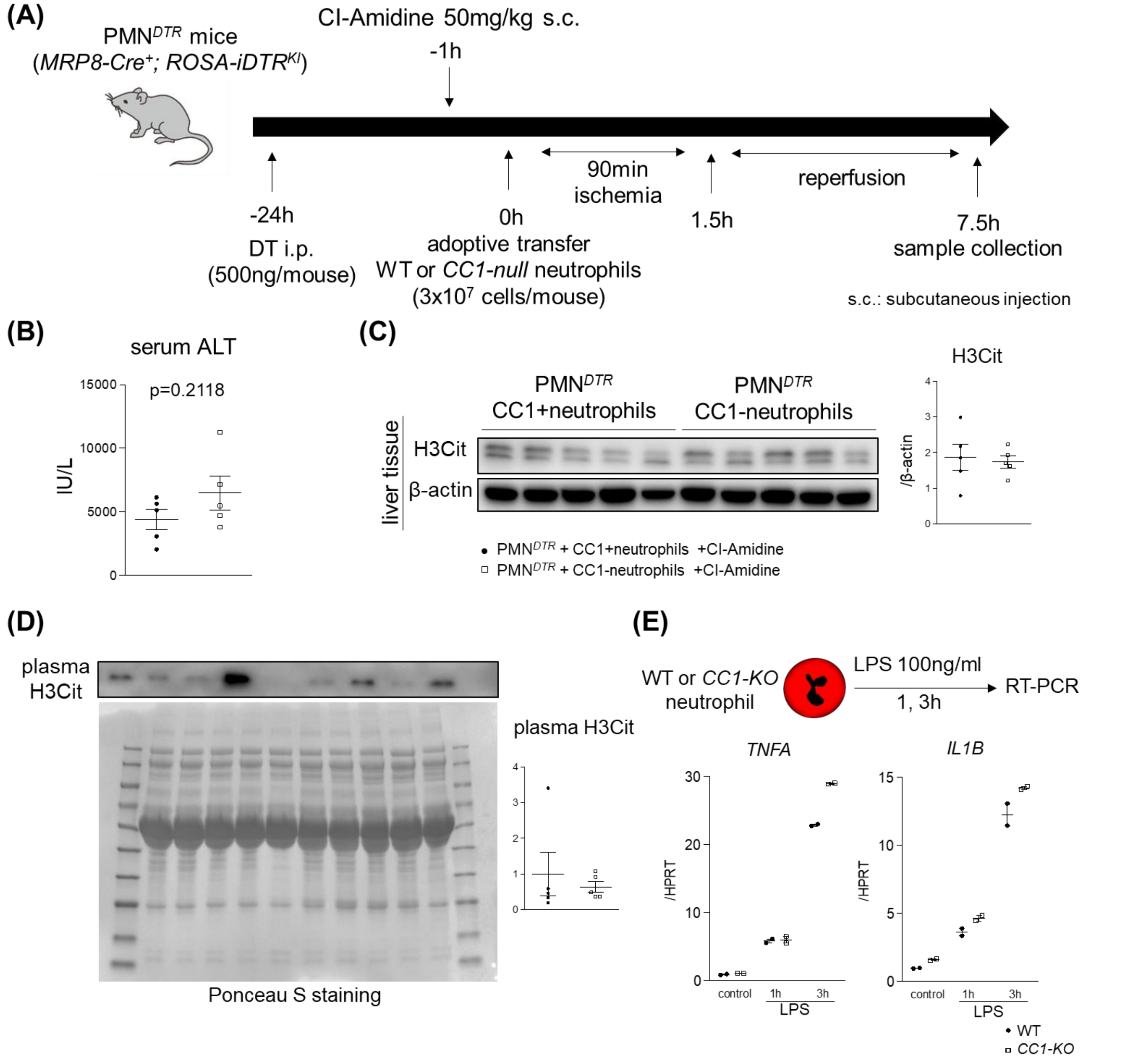

Suppl. Figure 10: IR injury in PMN^{DTR} mice repopulated with *CC1-null* neutrophils was mostly NETs-dependent: (A) Experimental scheme of IRI in PMN^{DTR} mice. **(B)** Serum ALT levels at 6h after reperfusion. Data shown as mean±SEM. **(C)** WB of H3Cit in post-IR livers. β-actin was used as an internal control. Data shown as mean±SEM. **(D)** WB of H3Cit in plasma samples and Ponceau S staining. Data shown as mean±SEM. **(E)** qRT-PCR-assisted detection of mRNA coding for TNFA and IL1B. Data, normalized to HPRT gene expression, shown as mean±SEM.

Figure S11

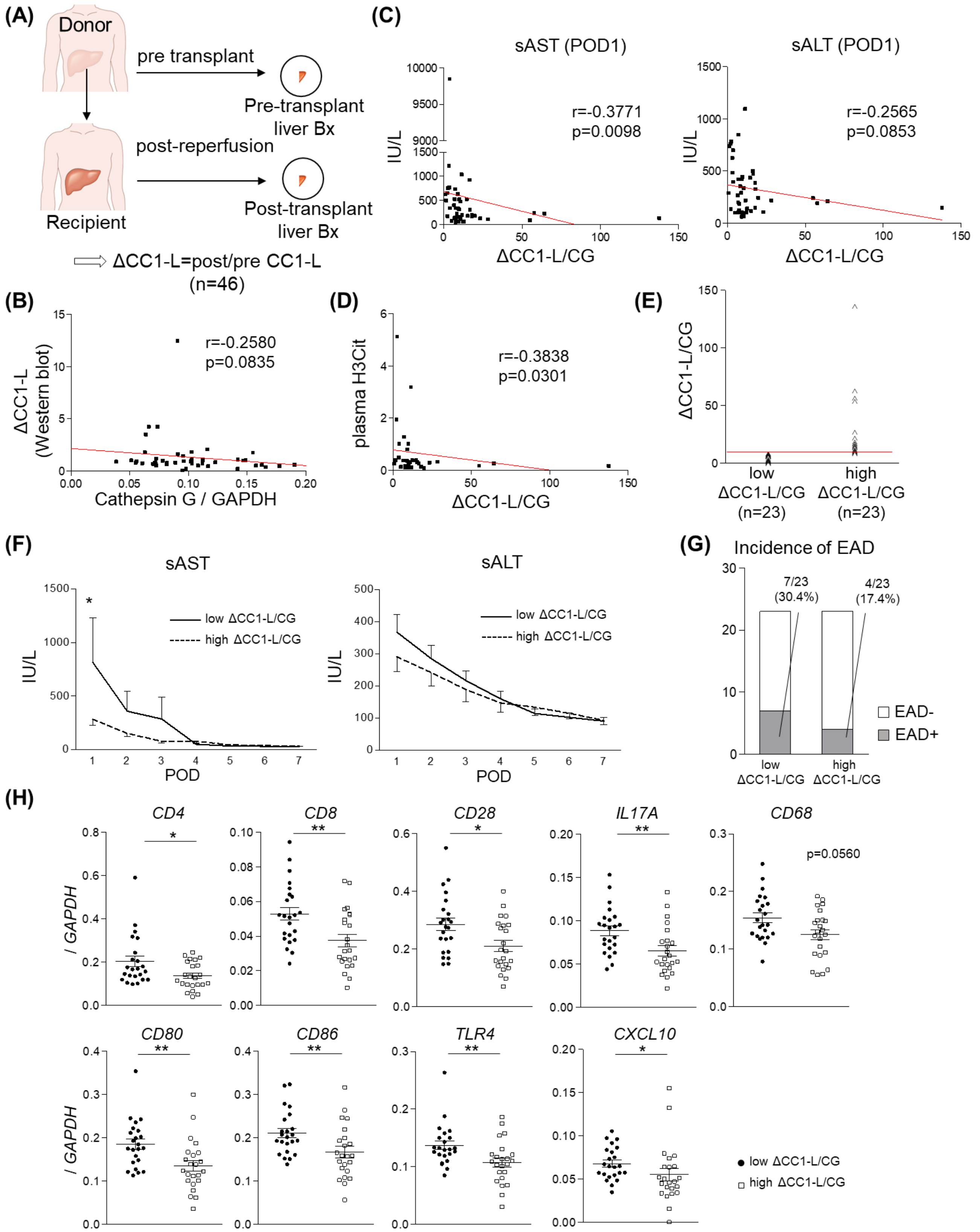

(A)
Donor
pre transplant
Pre-transplant liver Bx
post-reperfusion
Post-transplant liver Bx
Recipient
ΔCC1-L=post/pre CC1-L (n=46)
(B)
ΔCC1-L (Western blot)
r=-0.2580
p=0.0835
Cathepsin G / GAPDH
(C)
sAST (POD1)
r=-0.3771
p=0.0098
sALT (POD1)
r=-0.2565
p=0.0853
IU/L
ΔCC1-L/CG
(D)
plasma H3Cit
r=-0.3838
p=0.0301
ΔCC1-L/CG
(E)
ΔCC1-L/CG
low ΔCC1-L/CG (n=23)
high ΔCC1-L/CG (n=23)
(F)
sAST
sALT
IU/L
low ΔCC1-L/CG
high ΔCC1-L/CG
POD
(G)
Incidence of EAD
7/23 (30.4%)
4/23 (17.4%)
EAD-
EAD+
low ΔCC1-L/CG
high ΔCC1-L/CG
(H)
CD4
CD8
CD28
IL17A
CD68
p=0.0560
CD80
CD86
TLR4
CXCL10
/ GAPDH
low ΔCC1-L/CG
high ΔCC1-L/CG

Suppl. Figure 11: Increased peritransplant CC1-L/CathG levels correlate with attenuated hepatocellular damage and suppressed innate/adaptive immune responses in human OLT: (A) Pretransplant (post–cold storage) and posttransplant (2h after reperfusion) liver biopsies (Bx), collected from 46 OLT patients, were analyzed by WB with β-actin normalization for posttransplant/ pretransplant CC1-L ratios (ΔCC1-L). CathG levels were analyzed by qRT-PCR with normalization to GAPDH. **(B)** The relationship between ΔCC1-L and CathG. **(C)** The relationship between ΔCC1L/CathG and sAST/sALT at postoperative day 1 (POD1). **(D)** The relationship between ΔCC1-L/CathG and plasma H3Cit levels. r: Spearman correlation coefficient. **(E)** Human OLT biopsy samples were classified into low (n=23) and high (n=23) ΔCC1-L/CathG groups. **(F)** Serum AST and ALT levels at POD1-7 (*p<0.05, Mann-Whitney U test; data shown as mean±SEM. **(G)** Incidence of early allograft dysfunction (EAD) (Fisher's exact test). **(H)** qRT-PCR-assisted detection of mRNA levels coding for CD4, CD8, CD28, IL17, CD68, CD80, CD86, TLR4, and CXCL10. Data normalized to GAPDH gene expression are shown in dot plots and bars are indicative mean±SEM (* p<0.05, ** p<0.01, Mann-Whitney U test).

Figure S12

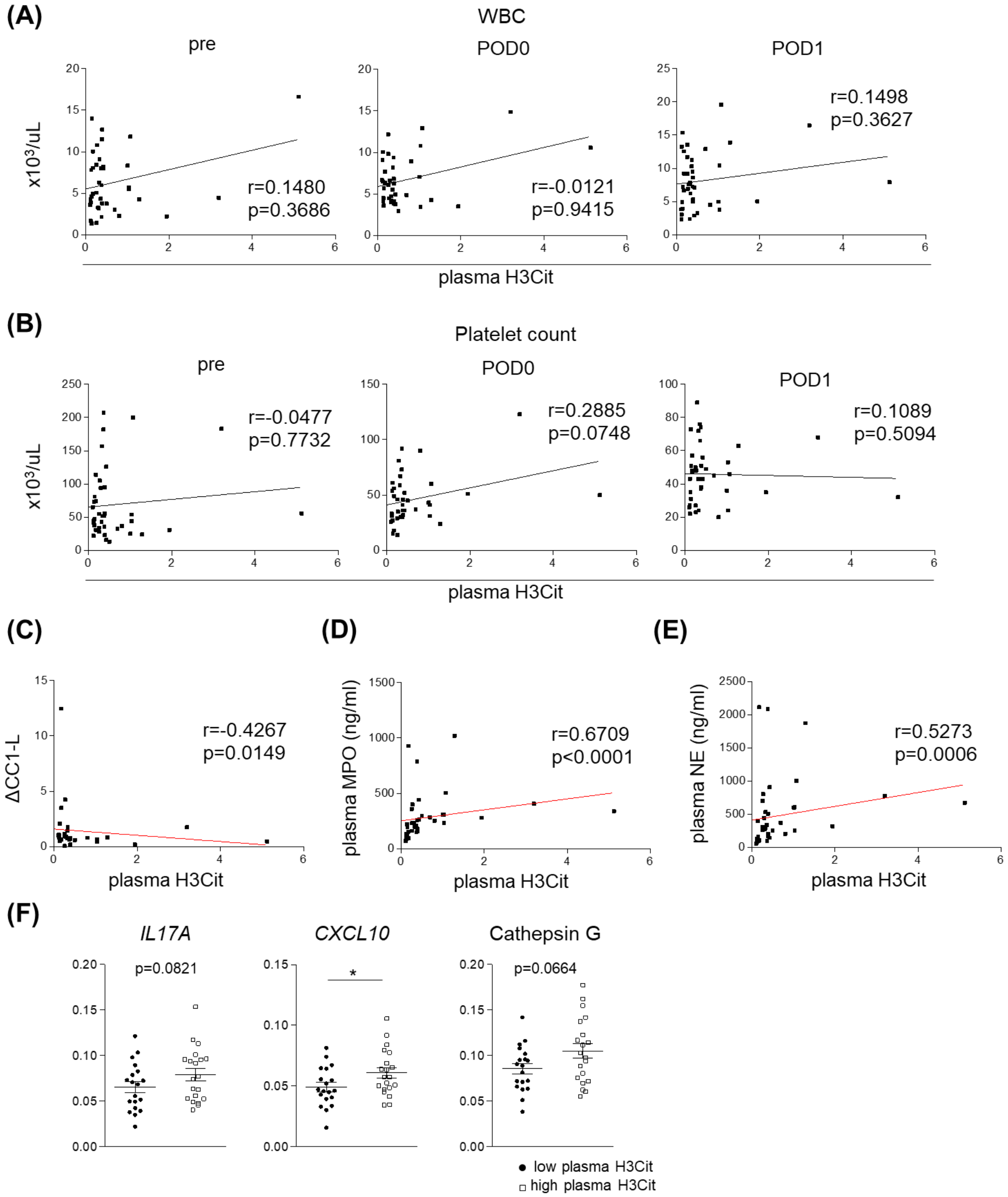

Suppl. Figure 12: Correlation between plasma H3Cit, peripheral blood profiles and graft inflammation in OLT patients: Relationship between plasma H3Cit levels and WBC **(A)** and platelet count **(B)** at pre-transplant, POD0 and POD1, △CC1-L level **(C)**, plasma MPO **(D)** and NE concentration **(E)**. **(F)** qRT-PCR-assisted detection of mRNA coding for IL17A, CXCL10 and Cathepsin G. Data normalized to GAPDH gene expression are shown in dot plots and bars indicative mean±SEM (* $p<0.05$, Mann-Whitney U test).

Figure S13

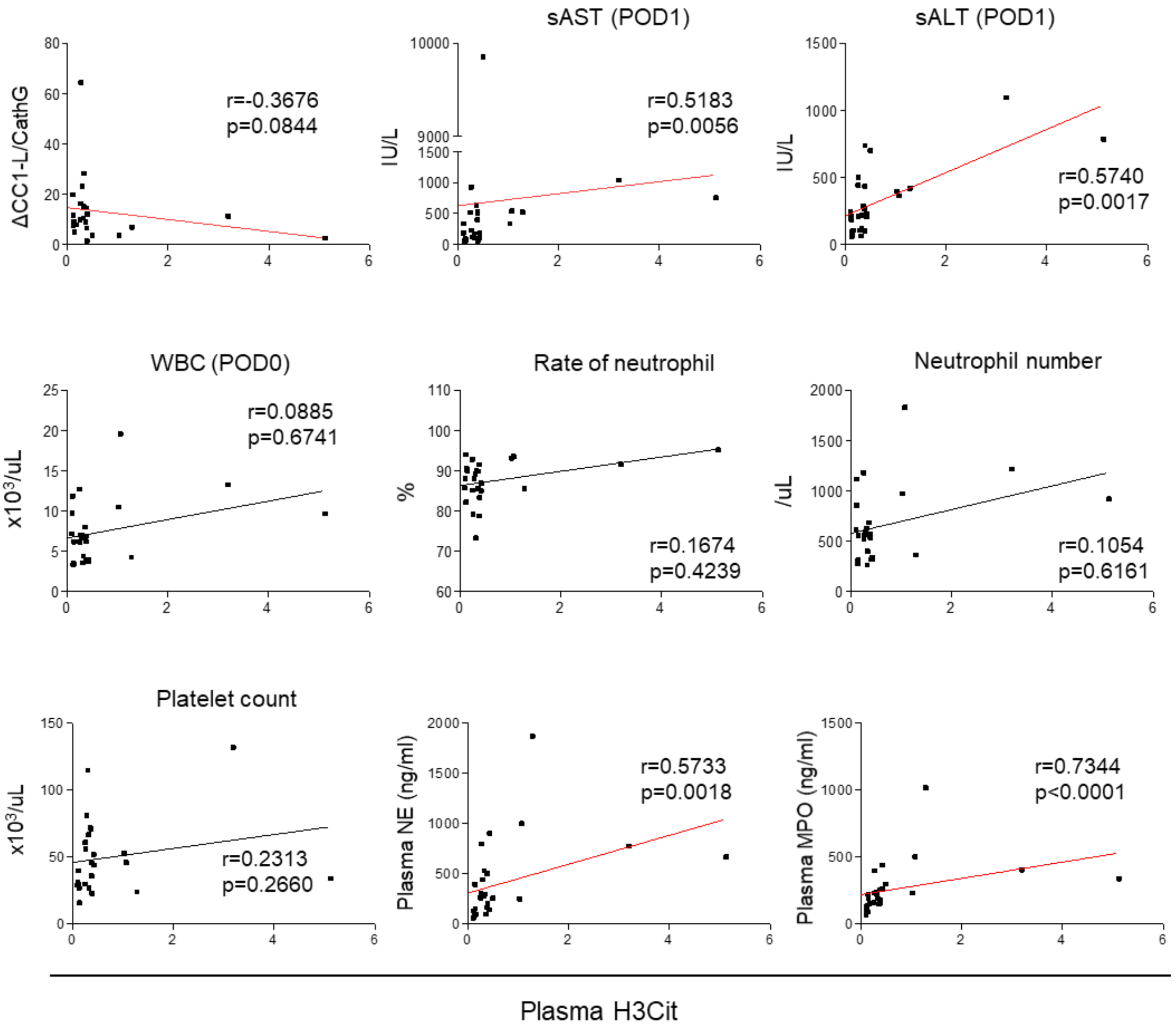

Suppl. Figure 13: Correlation between plasma H3Cit levels and Δ*CC1-L*/*CathG* ratio, transaminase release (POD1), peripheral blood parameter and plasma NE and MPO levels.

Figure S14

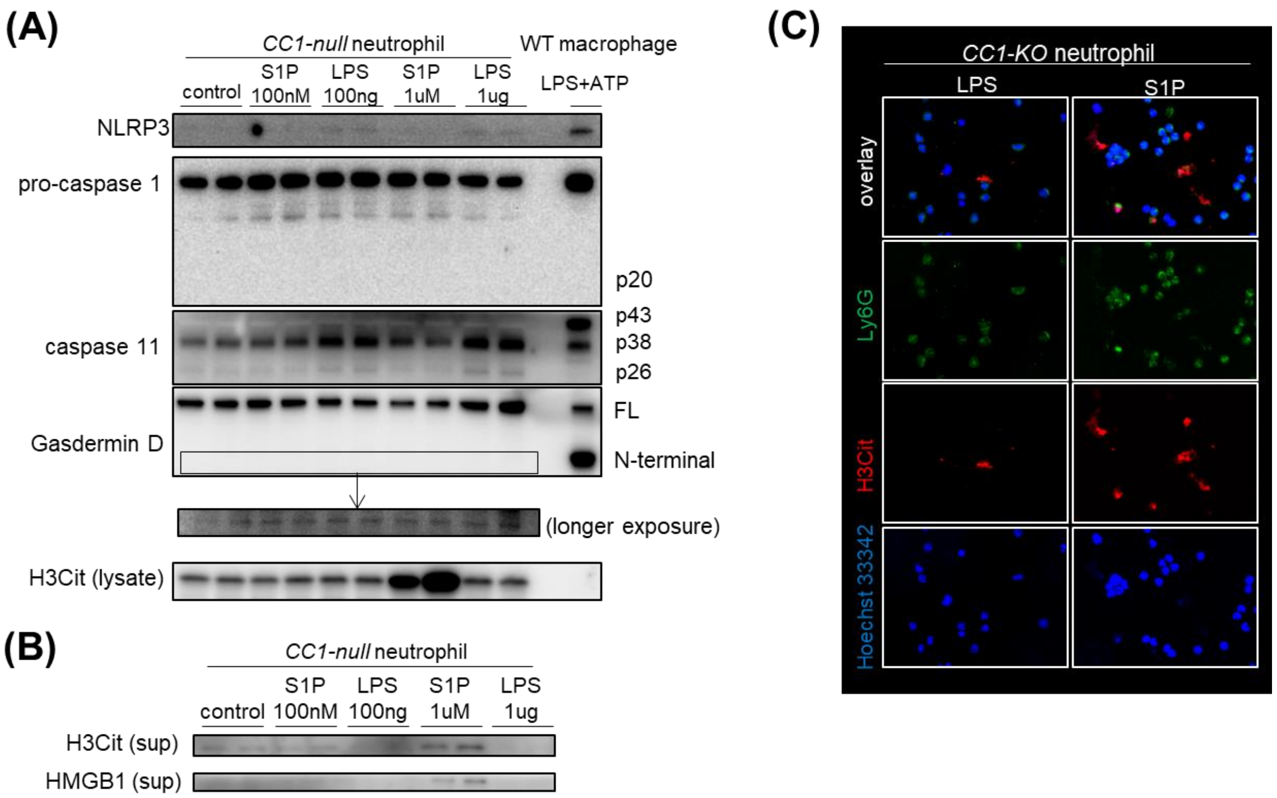

Suppl. Figure 14: S1P induces NETs independent of the inflammasome activation: (A) WB of NLRP3, caspase-1, caspase-11 and Gasdermin D (FL; full length of Gasdermin D) in CC1-deficient neutrophils treated with LPS (100ng or 1µg/ml, 4h) or S1P (100nM or 1µM, 4h). The lysate of WT macrophages stimulated with LPS (1µg/ml, 3h), followed by ATP (5mM, 30min), was used as a positive control for N-terminal of Gasdermin D. **(B)** WB of H3Cit and HMGB1 expression in the culture media of CC1KO neutrophils stimulated with LPS (100ng or 1µg/ml) or S1P (100nM or 1µM 4h). **(C)** Representative immunofluorescence of Ly6G (green), H3Cit (red) and Hoechst 33342 (blue) in CC1-KO neutrophils treated with S1P (1µM) or LPS (1µg/ml) for 4h (n=2/group).

Figure S15

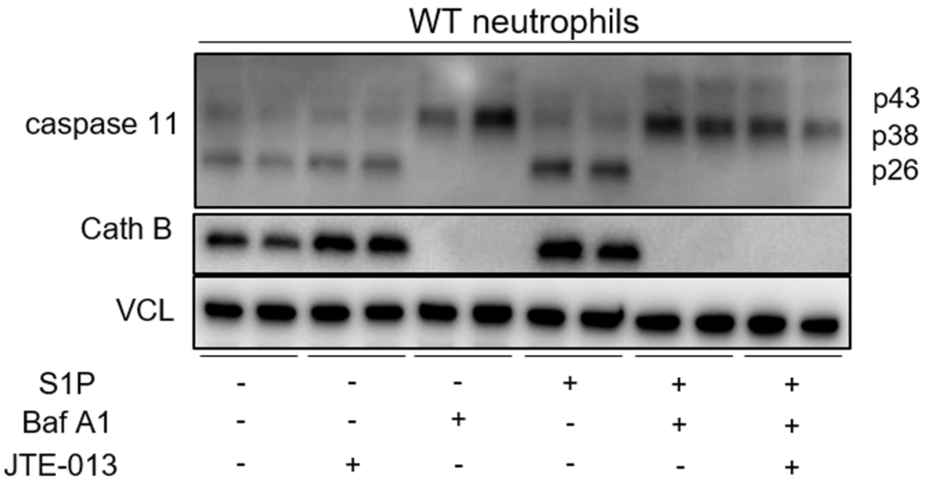

Suppl. Figure 15: Bafilomycin A1 inhibits the activation of caspase-11: WB-assisted detection of caspase-11, and Cathepsin B in WT neutrophils stimulated with JTE-013 (10µM, 0.5h); Baf A1 (100nM, 0.5h); or S1P (1µM, 4h). VCL was used as an internal control.

Supplemental Methods

Isolation of liver sinusoidal endothelial cells (LSEC) and Kupffer cells (KC)

Primary mouse non-parenchymal cells (NPCs) were isolated, as reported (1). LSEC and KC were isolated by magnetic-activated cell sorting (MACS) method according to the manufacturer protocol. CD146 (LSEC) MicroBeads (#130-092-007) or Anti-F4/80 MicroBeads UltraPure (#130-110-443, Bergisch Gladbach, Germany) were used.

Supplemental Results

S1P-induced NET formation is inflammasome activation-independent

We employed LPS to test whether NET formation induced by S1P activation was related to the inflammasome signaling pathway. S1P, but not LPS, stimulation augmented H3Cit expression (cell lysate and culture media) and HMGB1 release (culture media), as confirmed by WB (Suppl. Figure 3A/3B). However, WB-assisted detection of NLRP3, caspase-1, caspase11 and gasdermin revealed that inflammasome activation markers (NLRP3, caspase 11) were higher in LPS-treated neutrophils (Suppl. Figure 3A). In addition, DNA extrusion, assessed by immunofluorescence, was increased only by S1P stimulation (Suppl. Figure 3C). These results indicate S1P induces NET formation by a distinct mechanism from the inflammasome activation pathway.

Bafilomycin A1 treatment suppresses cathepsin B expression and caspase 11 activation

Neutrophils isolated from WT mice were pretreated with vehicle, JTE-013, Baf A1 or JTE-013 plus Baf A1, followed by S1P stimulation. Baf A1 treatment depleted cathepsin B and inhibited caspase 11 activation regardless of the adjunctive JTE-013 or S1P conditioning (Suppl. Figure 4). These results indicate Baf A1, but not S1P or JTE-013, directly suppresses the caspase 11 cleavage.

Supplemental Reference

1. Mohar I, Brempelis KJ, Murray SA, Ebrahimkhani MR, and Crispe IN. Isolation of Non-parenchymal Cells from the Mouse Liver. *Methods in molecular biology (Clifton, NJ).* 2015;1325(3-17.
